# ssODN-mediated knock-in with CRISPR-Cas for large genomic regions in zygotes

**DOI:** 10.1038/ncomms10431

**Published:** 2016-01-20

**Authors:** Kazuto Yoshimi, Yayoi Kunihiro, Takehito Kaneko, Hitoshi Nagahora, Birger Voigt, Tomoji Mashimo

**Affiliations:** 1 Institute of Laboratory Animals, Graduate School of Medicine, Kyoto University, Kyoto 606-8501, Japan; 2 Mouse Genomics Resource Laboratory, National Institute of Genetics, Shizuoka 411-8540, Japan; 3 Institute of Experimental Animal Sciences, Graduate School of Medicine, Osaka University, Osaka 565-0871, Japan; 4 BioDynamics Laboratory Inc., Tokyo 113-0033, Japan

## Abstract

The CRISPR-Cas system is a powerful tool for generating genetically modified animals; however, targeted knock-in (KI) via homologous recombination remains difficult in zygotes. Here we show efficient gene KI in rats by combining CRISPR-Cas with single-stranded oligodeoxynucleotides (ssODNs). First, a 1-kb ssODN co-injected with guide RNA (gRNA) and Cas9 messenger RNA produce GFP-KI at the rat *Thy1* locus. Then, two gRNAs with two 80-bp ssODNs direct efficient integration of a 5.5-kb CAG-GFP vector into the *Rosa26* locus via ssODN-mediated end joining. This protocol also achieves KI of a 200-kb BAC containing the human *SIRPA* locus, concomitantly knocking out the rat *Sirpa* gene. Finally, three gRNAs and two ssODNs replace 58-kb of the rat *Cyp2d* cluster with a 6.2-kb human *CYP2D6* gene. These ssODN-mediated KI protocols can be applied to any target site with any donor vector without the need to construct homology arms, thus simplifying genome engineering in living organisms.

## Abstract (web summary)

CRISPR-Cas9 is a powerful genome engineering tool but gene knock-in is limited by fragment size and efficiency of recombination. Here the authors used a modified strategy employing single-strand oligonucleotides to efficiently knock-in large DNA fragments and humanise native rat loci.

## Graphical abstract

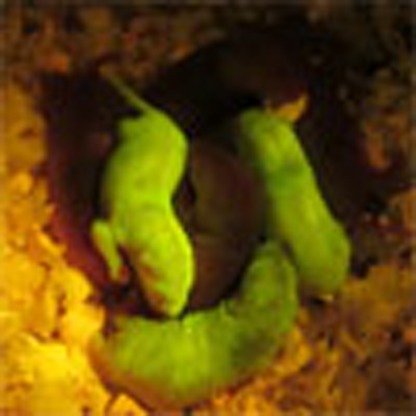

Genetically modified (GM) animals play an important role in the study of gene functions and for understanding the mechanisms of human diseases. Zinc finger nucleases, transcription activator-like effector nucleases (TALENs) and clustered regularly interspaced short palindromic repeats (CRISPR)-associated (Cas) nucleases are efficient genome engineering tools for generating GM animals via microinjection into fertilized eggs^1^,^2^,^3^,^4^,^5^,^6^. The injected nucleases recognize long stretches of DNA sequence and introduce DNA double-strand breaks (DSBs), which are generally repaired via non-homologous end-joining (NHEJ), a process that introduces small insertions or deletions (indels) at the repair junction. This process allows efficient generation of animals with knockout (KO) alleles at targeted sequences^7^,^8^,^9^. Targeted knock-in (KI) can be also engineered via homologous recombination (HR) by co-injection of donor plasmids, including large DNA fragments such as green fluorescent protein (GFP) cassettes, with any of the above mentioned nucleases^10^,^11^,^12^,^13^,^14^,^15^. This was traditionally achieved by constructing targeting vectors with two homology arms of 0.5–1 kb on either side of the insert DNA. However, HR-mediated KI is less efficient than NHEJ-mediated KO, because the canonical HR pathway for DSB repair in mammalian cells or embryos is less effective than the NHEJ pathway^16^,^17^,^18^.

Recently, single-stranded oligodeoxynucleotides (ssODNs) have been used as donor templates in combination with the engineered nucleases for efficient targeted insertion of small DNA fragments. ssODN-mediated KI in mammalian cells occurs via homology-directed repair (HDR) and is more efficient than using double-stranded donor plasmids^18^,^19^,^20^,^21^. In mice, targeted KI with ssODN donors and engineered nucleases has been reported to be highly efficient^22^,^23^,^24^,^25^. We have also shown the efficient generation of ssODN-mediated KI in rats by recovering three recessive coat-colour phenotypes^26^.

In this study, ssODNs in combination with methodical CRISPR-Cas fine tuning are employed to KI and replace large genomic regions of the rat genome with human genes. We first append polyadenine tails (poly(A)) to plasmids expressing Cas9 mRNA, to increase the efficacy of genome editing with the CRISPR-CasCRISPR-Cas system. Using Cas9-poly(A), we employ two approaches to conduct targeted KI with relatively long DNA fragments, such as the GFP sequence. The first use long ssODNs (lsODNs) as a targeting donor, which are newly synthesized by a method using nicking endonucleases. The second involve co-injection of two guide RNAs (gRNAs) to act as ‘scissors', to cut target sites in genomic DNA and the donor plasmid DNA, and two short ssODNs to act as ‘paste', to ligate the ends of the cut sites. This method enable efficient KI of plasmid DNAs including a bacterial artificial chromosome (BAC) of 200 kb. The significant technical advantage of this ssODN-mediated end joining approach is its simplicity, as there is no need to construct homology arms in the donor vector, which is especially difficult to perform in larger BACs.

## Results

### Poly-A tail elongation enhances genome editing efficiency

**Figure 1:**
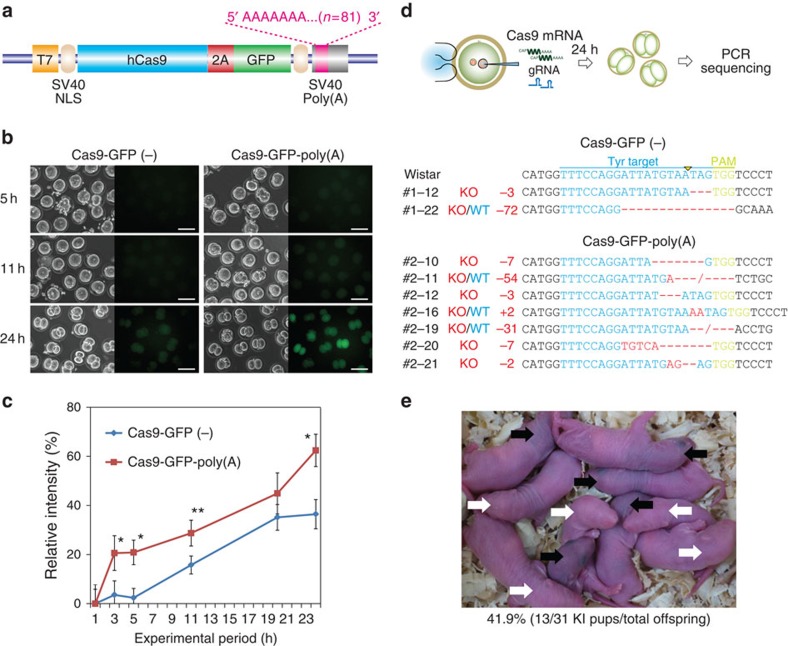
Poly(A) tail elongation enhances genome-editing efficiency in zygotes. (**a**) Schematic representation of the Cas9-2A-GFP plasmid. An 81-bp poly(A) was added to the 3′-untranslated region (UTR) of the construct. (**b**) *In vitro*-transcribed Cas9 mRNA (100 ng μl^−1^) was injected into pronuclei of rat fertilized eggs. Fluorescence microscopy images were captured after injection. (**c**) The fluorescence intensity of oocytes was estimated as relative intensity with the highest intensity of oocytes after 24-h incubation set at 100%. *P<0.05 and **P<0.001 by Student's *t*-test. Error bars represents.e.m. (**d**) Co-injection of gRNA (50 ng μl^−1^) targeting the rat tyrosinase (*Tyr*) gene with Cas9 mRNA into rat fertilized eggs resulted in NHEJ-mediated KO mutations at the targeted sites (Supplementary Fig. 1). Two-cell embryos were collected and genomic DNA was amplified by PCR to detect NHEJ-mediated KO mutations at the targeted *Tyr* locus. (**e**) Co-injection of ssODN (50 ng μl^−1^) with gRNA and Cas9-poly(A) mRNA into rat fertilized eggs corrected the albino phenotype (white arrows) by HDR-mediated single nucleotide polymorphism (SNP) exchange, to produce the black-hooded phenotype (black arrows).

Poly(A) tails play an important role in the stability and the translation of most eukaryotic mRNAs^27^,^28^,^29^. To increase the translation efficiency of the Cas9 mRNA in zygotes, we appended an 81-bp poly(A) signal to the transcription terminator of the Cas9-2A-GFP plasmid (Addgene ID#44719) and injected *in vitro*-transcribed Cas9 mRNA (100 ng μl^−1^) into rat male pronuclei of fertilized eggs (Fig. 1a). Three hours after injection, eggs treated with Cas9-GFP-poly(A) (*n*=23) showed significantly higher fluorescence intensity compared with eggs injected with Cas9-GFP without poly(A) (*n*=20) (Fig. 1b,c). The higher expression in the Cas9-GFP-poly(A)-injected eggs persisted at the two-cell stage 24 h after injection (Fig. 1b,c). In addition, eggs injected with Cas9-GFP-poly(A) did not show any significant abnormal features or delayed development compared with eggs without poly(A) during the 24-h culture period. These findings indicate that an elongated poly(A) tail can enhance the translation of Cas9 mRNA in rat embryos.

Next, we co-injected 50 ng μl^−1^ gRNA and Cas9-GFP-poly(A) mRNA targeting the rat Tyrosinase (*Tyr*) gene^26^ into fertilized rat eggs (Supplementary Table 1). After 24 h, two-cell embryos were collected and genomic DNA was amplified to analyse NHEJ-mediated KO mutations at the targeted *Tyr* locus (Fig. 1d). Cas9-poly(A)-injected embryos showed a higher rate of NHEJ-mediated indel mutations (7/19, 36.8%) compared with wild-type Cas9-injected embryos (2/20, 10.0%) (Fisher's exact test: *P*<0.05; Supplementary Table 1).

We then co-injected 50 ng μl^−1^ ssODNs with gRNA and Cas9-poly(A) mRNA (without GFP) into fertilized rat eggs, to recover the albino phenotype (a missense mutation in *Tyr*) by ssODN-mediated single nucleotide polymorphism exchange, as previously described^26^. Transfer of the injected embryos into pseudopregnant female rats resulted in the birth of 31 pups, among which 20 (64.5%) showed NHEJ-mediated KO mutations and 13 (41.9%) showed HDR-mediated KI mutations, indicated by phenotype recovery from albino to black hooded (Fig. 1e, Table 1 and Supplementary Fig. 1). Among the 13 animals with a KI mutation, 7 also showed NHEJ-mediated indels. These mutation rates for KO and KI by Cas9-poly(A) are significantly higher than the 23.1% KO and 7.7% KI found in our previous study without poly(A) enhancement^26^ (Fisher's exact test: *P*<0.005 for KO and *P*<0.05 for KI). Neither insertions nor deletions were observed in the 13 KI rats at any off-target sites across the whole genome, examined according to standard criteria^26^ (Supplementary Table 2).

### GFP KI at the rat *Thy1* locus using long ssODNs

**Figure 2:**
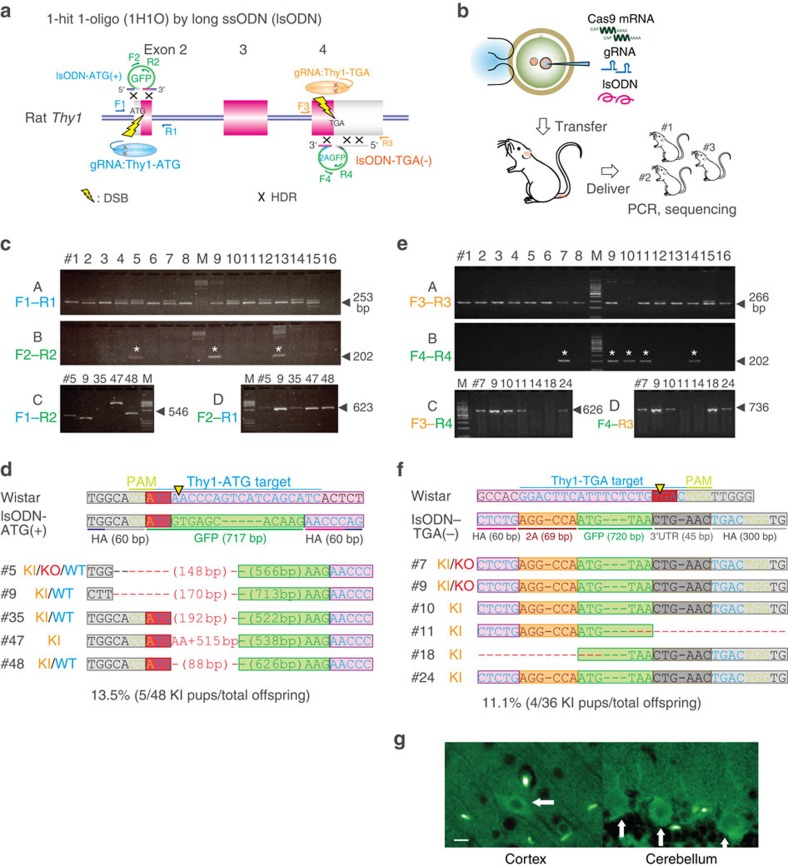
lsODNs with CRISPR-Cas mediates KI at the rat *Thy1* locus. (**a**) Schematic representation of the rat thymus cell antigen 1 (*Thy1*) gene, gRNA targeting sites (Thy1-ATG and Thy1-TGA), primer sets (F1–4 and R1–4) and the lsODNs, each containing a GFP cassette and two 60–300 bp homology sequences. (**b**) Schematic of gRNA, Cas9 mRNA and lsODN co-injection into rat fertilized eggs to generate founders carrying the KI mutation. (**c**) PCR analysis of pups (1–16) derived from co-injection of gRNA:Thy1-ATG, Cas9 mRNA and lsODN-ATG(+) with primer sets, F1,2 and R1,2, as indicated in **a** (see also Supplementary Table 3). Many pups show indel mutations at the targeted Thy1-ATG locus (also shown in Supplementary Fig. 4). (A) The product size of the wild-type Wistar allele (arrowed) is 253-bp. Some pups showed a GFP-positive PCR band (asterisk). (B) PCR-positive band with primer sets, F1 and R2 (C) or F2 and R1 (D) indicate ligation at either side of the Thy1-ATG and the GFP. Various indel mutations were found in c, but not in d, after comparison with the predicted PCR size indicated by arrows. (**d**) Sequence analysis of the GFP-positive pups showed HDR-mediated KI alleles with partial deletions of 5′-GFP coding sequences. (**e**) Co-injection of gRNA:Thy1-TGA, Cas9 mRNA and the complementary lsODN(−) with extended 300-bp homology arms into rat fertilized eggs. Many pups showed indel mutations at the targeted Thy1-TGA locus (Supplementary Fig. 6). (A) The product size of the wild-type Wistar allele (arrowed) is 266 bp. Some pups showed a GFP-positive PCR band (asterisk). (B) PCR analysis of the pups with primer sets F3 and R4 (C) or F4 and R3 (D) showed the ligation at either side of Thy1-TGA and GFP. (**f**) Sequence analysis of 7 GFP-positive pups showed that four rats (numbers 7, 9, 10 and 24) carried complete sequences of HDR-mediated KI alleles, including rat Thy1, 2A peptide and GFP. The remaining three failed to be sequenced at the conjunction sites. (**g**) KI rat number 24 showed GFP-positive neurons in the cortex and GFP-positive Purkinje cells in the cerebellum (arrows). Blood vessels were also GFP positive. Scale bar, 10 μm.

We have previously used the CRISPR-Cas system together with a 119-bp ssODN, to accomplish targeted integration of a 19-bp nucleotide fragment at the agouti signalling protein locus (*Asip*) to recover the non-agouti phenotype^26^. To integrate longer DNA fragments, such as a GFP reporter cassette, we synthesized an 837-bp lsODN consisting of GFP-coding sequences and two 60-bp homology arms at either end of the GFP sequence (Fig. 2a and Supplementary Fig. 2). We then designed gRNA targeting the start codon (ATG) of the rat thymus cell antigen 1 locus (*Thy1*) to produce THY1-GFP fusion protein. Microinjection of gRNA:Thy1-ATG, Cas9-poly(A) mRNA, together with 50 ng μl^−1^ lsODN into Wistar rat zygotes resulted in the birth of 48 pups (Fig. 2b, Supplementary Fig. 3 and Supplementary Table 3). PCR and subsequent sequence analysis revealed that 37 pups (77.1%) showed NHEJ-mediated KO mutations and 5 pups (13.5%) carried HDR-mediated KI sequences at the targeted site (Fig. 2c and Supplementary Fig. 4). However, all KI alleles included partial insertion or deletion at the 5′-region of the *Thy1* gene, which prevented translation of the complete GFP-THY1 fusion protein (Fig. 2d).

As the observed deletion mutations on the 5′-side of the integrated lsODN sequences were probably caused by the 5′–3′ exonuclease activity of the DSB repair system^18^ (see Discussion), we targeted the stop codon (TGA) of the rat *Thy1* gene with a complementary lsODN(−), which included the 2A peptide, GFP and 3′-untranslated region sequences, as well as an extended 5′-homology arm of 300 bp, to avoid the effect of possible exonuclease activity (Fig. 2a and Supplementary Fig. 5). We obtained 36 pups, of which 33 (91.7%) showed KO mutations (Supplementary Fig. 6) and 7 showed GFP-positive alleles (Fig. 2e). Among these 7 pups, two (numbers 11 and 18) were positive for a PCR product amplified across 1 of the *Thy1*-GFP junction sequences and 4 pups (11.1%, numbers 7, 9, 10 and 24) were positive for PCR products amplified across both of the junction sequences. Sequence analysis revealed that all four pups carried entire sequences for rat *Thy1*, the 2A peptide and GFP (Fig. 2f). Finally, in these pups, GFP fluorescence was observed in brain sections of cortical neurons and cerebellar Purkinje cells (Fig. 2g).

### CAG-GFP plasmid KI at the *Rosa26* locus

**Figure 3:**
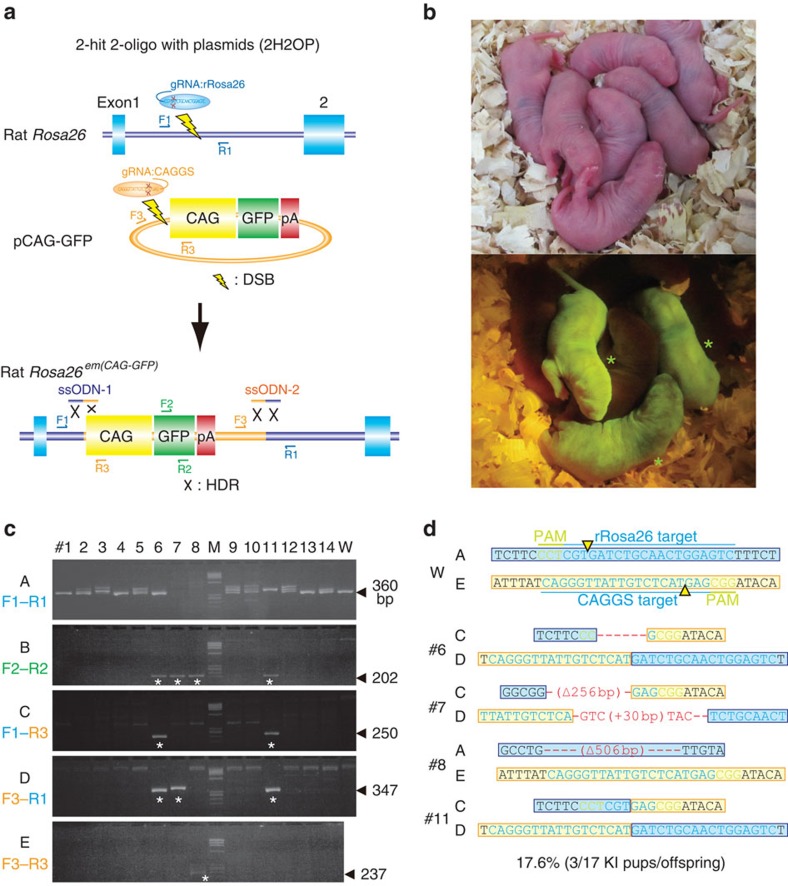
CAG-GFP KI rats generated using the CRISPR-Cas system. (**a**) Schematic representation of the two-hit two-oligo with plasmid (2H2OP) method. In the first step, Cas9, together with two gRNAs targeting the rat *Rosa26* locus and the CAG promoter in the GFP plasmids, cut the target sites. In the second step, two ssODNs ligate each cut end to join the genomic DNA and the plasmid DNA via HDR. (**b**) Photos of pups delivered from Wistar rat embryos injected with a mix of Cas9-poly(A) mRNA, two gRNAs, two ssODNs and CAG-GFP plasmids (upper). Some of the pups (numbers 6, 7 and 8) expressed GFP at a high level in the body (asterisk) (lower). (**c**) PCR analysis of the pups with primer sets F1–3 and R1–3, as indicated in **a**. Many pups showed indel mutations at the targeted *Rosa26* locus, compared with the 360-bp wild-type allele indicated by arrows (Supplementary Fig. 9) (A). Four pups showed a GFP-positive PCR band (asterisk) (B). Three pups, numbers 6, 7 and 11, were positive for the ligation between either side of *Rosa26* and CAG-GFP (C,D). Rat number 8 was positive for the uncut CAG sequences (E). Arrows indicate the predicted PCR size. (**d**) Sequence analysis of pups indicated that rat number 11 carried accurate conjunction at both cut ends.

In recent times, we have successfully deleted a 7-kb fragment of endogenous retroviral DNA from the genome of coat-colour-modified rats^26^. The experiment was designed with two gRNAs to cut on either side of the retroviral insertion and one ssODN that included homology arms to link the two DSBs of the original rat DNA. Here we applied a similar design to knock in longer plasmids, including the 1.2-kb CAG promoter and a 0.8-kb GFP cassette sequence (Supplementary Fig. 7). Following the previously reported NHEJ-dependent KI strategy^30^, we constructed two gRNAs: one targeting the rat *Rosa26* locus to cleave genomic DNA (Supplementary Fig. 8) and the other targeting 5′ of the CAG promoter sequence for concurrent cleavage of the plasmid DNA. We also designed two 80-bp ssODNs to ligate the two cut ends, as shown in Fig. 3a. We named this strategy ‘two-hit by gRNA and two oligos with a targeting plasmid' (2H2OP). We co-injected a mix of 100 ng μl^−1^ of the Cas9-poly(A) mRNA, 50 ng μl^−1^ of each of the two gRNAs, 50 ng μl^−1^ of each of the two ssODNs and 5 ng μl^−1^ of the CAG-GFP plasmid into Wistar rat embryos (Table 1). Of the 17 pups delivered, 4 expressed GFP in the whole body (Fig. 3b and Supplementary Fig. 9). PCR analysis showed specific amplification of GFP sequences in 4 of the 17 pups (numbers 6, 7, 8 and 11) (Fig. 3c). Primer sets designed for either side of each *Rosa26* and CAG-GFP junction (F1–R3 or F3–R1 in Fig. 3a) amplified the junction sequences in pups numbers 6, 7 and 11. However, a primer set designed to amplify across the cleavage site of the CAG-GFP plasmid produced a PCR product in pup number 8 (Fig. 3c). Sequence analysis of the 4 pups revealed that number 11 had accurate conjunction at both of the 2 cut ends, number 6 had a 6-bp deletion on one side and number 7 had a several-base-pair deletion and insertion on both sides (Fig. 3d).

Crossing rat numbers 8 and 11 with Wistar rats resulted in the faithful transmission of the GFP allele to the next generation (Supplementary Table 4). Again, no off-target effect was observed at any candidate sites in these rats (Supplementary Table 2). Southern blot analysis confirmed that rat number 11 had a single-copy GFP KI allele at the *Rosa26* locus, and that rat number 8 had an allele of three GFP transgene copies inserted at a random chromosomal site (Supplementary Fig. 10). Fluorescent *in situ* hybridization (FISH) analysis using labelled CAG-GFP probes in homozygous offspring from rat number 11 (W-*Rosa26*^*em1(CAG-GFP)Kyo*^, which are deposited in the National BioResource Project for the rat (NBRP-Rat): No. 0770) confirmed that the KI allele was integrated into rat chromosome 4q41-q42 where *Rosa26* is located (Supplementary Fig. 11). Heterozygous W-*Rosa26*^*em1(CAG-GFP)Kyo*^ rats showed intense GFP fluorescence from only one copy of the KI allele (Supplementary Fig. 12). They also showed uniform expression in most cells and tissues examined, such as the brain, liver, heart and pancreas (Supplementary Fig. 13).

Considering the repair system for CRISPR-mediated DSBs with ssODNs, where 5′–3′ exonuclease activity may cause indel mutations, we again used a complementary ssODN(−) upstream of the *Rosa26* cut site (Supplementary Fig. 14a). We observed that the complementary ssODN(−) increased the KI efficiency slightly but not significantly (28.6%; 6/21; Supplementary Table 5), although 5 of 6 KI rats still showed several base-pair deletions or insertions on either side of the KI (Supplementary Fig. 14b). This 2H2OP approach was further applied to C57BL/6J mice, to knock in CAG-GFP at the mouse *Rosa26* locus (Supplementary Fig. 15). Of the 31 pups that were delivered, all 31 (100%) showed indel mutations at the mouse *Rosa26* locus (Supplementary Fig. 16). Sequence analysis revealed that three pups (9.7%) expressing GFP in the whole body carried the KI allele with conjunct sequences between *Rosa26* and the CAG-GFP plasmid (Supplementary Fig. 17).

### Human BAC KI at the rat *Sirpa* locus

**Figure 4:**
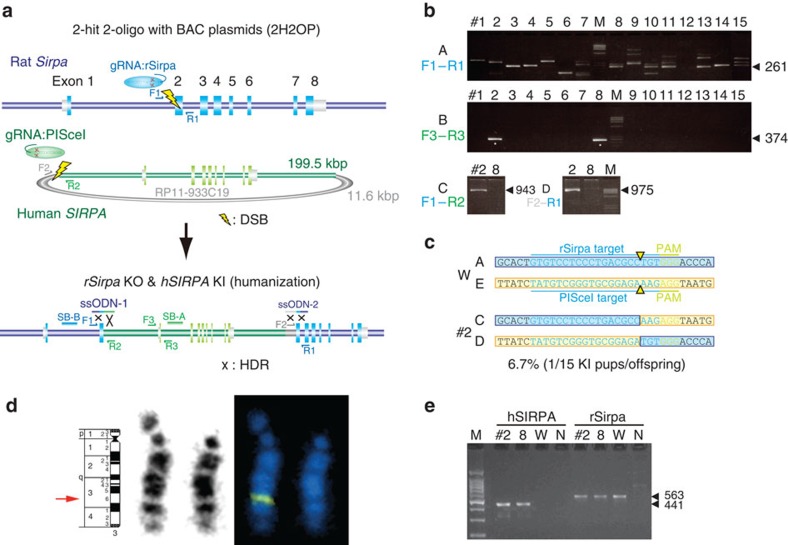
Human BAC KI at the *SIRPA* locus. (**a**) Schematic representation of the BAC KI by 2H2OP. The rat *Sirpa* gene was knocked out by KI of the 200-kb BAC (RP11-933C19), which contains the human *SIRPA* locus. (**b**) PCR analysis of pups with primer sets F1–3 and R1–3, as indicated in **a**. Many pups showed indel mutations at the targeted rat *Sirpa* locus compared with the 261-bp wild-type allele (arrow) (A) and two pups showed a human *SIRPA*-positive PCR band (asterisk) (B). Rat number 2 was positive for ligation between both sides of rat *Sirpa* and human *SIRPA* (C,D). Arrows indicated the predicted PCR fragment sizes. (**c**) Sequence analysis of pups indicated that rat number 2 carried accurate conjunction at both cut ends. (**d**) The heterozygous *SIRPA*-KI allele was confirmed by FISH on rat chromosome 3q35 (arrow). (**e**) Reverse transcriptase–PCR analysis of human SIRPA mRNA in the liver of rat numbers 2 and 8. N, negative control; W, control Wistar.

To investigate whether much larger plasmids, such as BAC clones, could be knocked in to a targeted genomic region, we used the 2H2OP approach to knock out the rat *Sirpa* gene and concomitantly knock in the human *SIRPA* gene, which is contained within the 200-kb BAC clone, RP11-933C19 (Fig. 4a). Cas9 mRNA, two gRNAs targeting exon 2 of rat *Sirpa* (Supplementary Fig. 18) and the PI-SceI site of pBACe3.6 (Supplementary Fig. 19), and two ssODNs for ligation of the rat genome sequences and the 933C19 BAC sequence, were injected into Wistar rat embryos. We obtained 15 pups from 64 embryos injected (Table 1). PCR analysis revealed that 13 pups (86.7%) carried KO alleles at the rat *Sirpa* locus (Supplementary Fig. 20), and that 2 pups (13.3%) were positive for the human *SIRPA* gene (Fig. 4b). Primer sets designed to amplify across each side of the rat *Sirpa* locus and PI-SceI junction (F1-R3 or F3-R1 in Fig. 4a) amplified the conjunct sequences in rat number 2 (Fig. 4b). Sequence analysis revealed that rat number 2 had accurate conjunction at both of the two cut ends (Fig. 4c).

To examine whether the complete 200-kb sequence or only a part of it was inserted at the targeted site, we performed PCR analysis using primer sets amplifying the whole human *SIRPA* gene and BAC vector sequences, all of which were positive for KI rat number 2 (Supplementary Fig. 21). FISH analysis of rat KI number 2 using labelled BAC vector probes revealed that the KI allele was integrated into rat chromosome 7q41-q42 where Sirpa is located (Fig. 4d). Finally, reverse transcriptase–PCR using primer sets amplifying hSIRPA or rSirpa complementary DNA showed unique expression of the human *SIRPA* gene in the KI rats (Fig. 4e).

### Genomic humanization of the *Cyp2d* cluster genes

**Figure 5:**
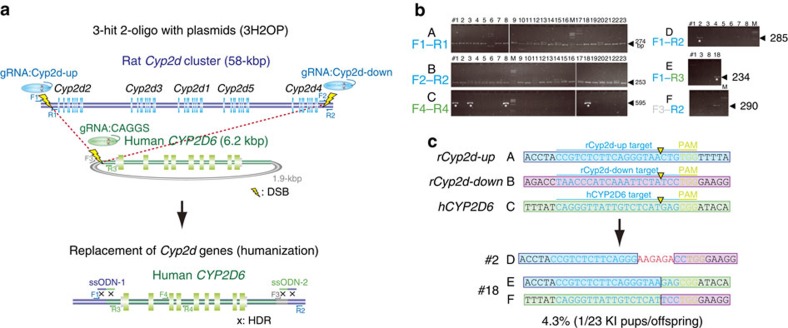
Replacement of the rat *Cyp2d* cluster with the human *CYP2D6* gene. (**a**) Schematic representation of the 3H2OP method to replace the rat *Cyp2d* cluster genes, *Cyp2d1–5* (58 kb), with the orthologous human *CYP2D6* gene (6.2 kb). Three gRNAs for upstream and downstream of the rat *Cyp2d* cluster and for the human *CYP2D6* plasmid cut the targeting sites, and two ssODNs ligate to each cut end. (**b**) PCR analysis of pups with primer sets F1–4 and R1–4 as indicated in **a**. Many pups showed indel mutations compared with the wild-type alleles upstream (274 bp) and downstream (253 bp) of *Cyp2d*, indicated by arrows (A and B, respectively). Four pups showed a positive PCR band for the human *CYP2D6* gene (asterisk) (C). Rat number 18 was positive for ligation between both sides of the rat *Cyp2d* cluster and human *CYP2D6* (D,E). Rat number 2 showed a large deletion from upstream to downstream of the rat *Cyp2d* cluster (E). Arrows indicate the predicted PCR fragment size. (**c**) Sequence analysis of pups indicated that rat number 18 carried the accurate conjunction at both cut ends.

Although human cytochrome P450 2D6 (CYP2D6) is one of the most important enzymes involved in the metabolism of xenobiotics, in particular clinically used drugs, the orthologous rat gene remains unknown^31^,^32^. However, the gene encoding CYP2D6 is assumed to be located in the rat *Cyp2d1–5* gene cluster. To delete the whole 58-kb region containing five orthologous rat genes, *Cyp2d1–5*, and to knock in a 6.2-kb sequence of the human *CYP2D6* gene, we designed three gRNAs: one targeting upstream of the rat *Cyp2d2* locus, the second downstream of the rat *Cyp2d4* locus (Supplementary Fig. 22) and the third targeting the plasmid that contains human *CYP2D6* (Fig. 5a). We then co-injected two 120-bp ssODNs together with the *CYP2D6* plasmid, to ligate each cut end of the rat *Cyp2d1–5* cluster region with the cut ends of the human *CYP2D6* plasmid. We obtained 23 pups delivered from 72 transferred embryos (Table 1 and Supplementary Fig. 23). PCR analysis with primer sets for the human *CYP2D6* locus and the rat *Cyp2d* loci indicated that rat numbers 1, 3, 8 and 18 carried the human *CYP2D6* gene, and that rat number 2 carried the deletion allele of the whole rat *Cyp2d* cluster (primer sets C and set D, respectively in Fig. 5b). Primer sets E and F, designed to amplify either end of the of the human *CYP2D6* insertion into the rat *Cyp2d* cluster, amplified the conjunct sequences in rat number 18, indicating that gene(s) replacement of the rat *Cyp2d* cluster with the human *CYP2D6* gene was accomplished by the 3-hit 2-oligo with plasmids (3H2OP) method (Fig. 5b). The replacement was confirmed by sequence analysis as shown in Fig. 5c. FISH analysis of the number 18 KI rat using labelled plasmid probes revealed that the human *CYP2D6* allele was integrated into rat chromosome 7q34 where the rat *Cyp2d* is located (Supplementary Fig. 24).

## Discussion

Plasmids consisting of homology arms, the targeting cassette and antibiotic selection markers are generally used for targeted KI via HR in mammalian cells, including embryonic stem and induced pluripotent stem cells. The recent combination of this approach with site-specific endonucleases, such as CRISPR-Cas, drastically increased targeting efficiency in these cultured cells^33^,^34^,^35^. In zygotes, targeted KI with plasmids via classical HR has also been reported by several groups^13^,^14^,^15^,^24^. However, the efficiency is still not ideal, because selection markers are not available. In this study, we have shown two new approaches that use the CRISPR-Cas system. The first used a synthetic long ssODN containing a GFP cassette and successfully knocked in GFP at the 3′-end of the rat *Thy1* gene. The second approach, 2H2OP, used two short ssODNs for the integration of a longer plasmid, which allowed the efficient integration of a CAG-GFP vector into the rat *Rosa26* locus. In addition, 2H2OP allowed KI of a 200-kb BAC containing the human *SIRPA* gene at the rat *Sirpa* locus and also achieved replacement of the rat *Cyp2d* cluster genes with the human *CYP2D6* gene. To the best of our knowledge, this is the first report of the targeted KI of such a long BAC fragment and the replacement of a large genomic region containing a cluster of genes by a single injection of unmodified donor DNA, ssODNs and CRISPR-Cas into rodent embryos.

Recent studies showed the high efficiency of CRISPR-Cas-mediated KI in zebrafish by concurrent cleavage of donor plasmid DNA and genomic DNA using an NHEJ-dependent DNA repair system^36^,^37^. More recently, microhomology-mediated end-joining-dependent targeted KI of donor plasmid DNA by TALEN or CRISPR-Cas was reported as TALEN-mediated precise integration into target chromosome system (PITCh) or CRISPR-mediated PITCh methods respectively, in human cells and frogs^38^. These KI technologies are both achieved by the concurrent cleavage of genomic DNA and donor plasmids without using ssODN, but have not been used in mouse and rat zygotes. As shown in Table 1, we injected Platinum TALENs^39^ into rat zygotes targeting the *Rosa26* locus with a TALEN-mediated-PITCh vector containing CAG-GFP sequences, resulting in several TALEN-mediated KO pups (9/32), but no pups with GFP KI alleles (0/32) at the *Rosa26* locus. The apparent difference in KI efficiency between fish–frogs and rodents might reflect the different dependence on the DNA repair system between species. In contrast, the 2H2OP method in this study provided several pups with GFP KI alleles (3/17) at the same *Rosa26* locus, suggesting that co-injected ssODNs can support the ligation of CRISPR-Cas-mediated DSBs of the template into genomic target DNA. The increased ligation efficiency via ssODN-mediated end joining was also observed in our previous studies^26^.

Several groups have reported HR-dependent KI in rats using targeting vectors and zinc finger nuclease^10^, TALEN^13^,^40^ and CRISPR^14^,^15^. The efficiency of HR-mediated KI is strongly dependent on the gene being targeted, but technical conditions are also a factor. Ma *et al.*^14^ reported >50% KI efficiency (6/11 pups) at the rat *Cck* locus; however, most other studies have reported a lower efficiency (1–30%) or failure of KI at certain genes. In our experiments, co-injection of Cas9-polyA, gRNA targeting *Rosa26* and a donor plasmid comprising CAG-GFP sequences flanking 515 and 645-bp homology arms into rat zygotes resulted in no pups with KI alleles (0/25; Table 1). Homology arms are usually cloned into targeting vectors for HR-based experiments, whereas no homology arms are required in existing plasmids in 2H2OP. The notable advantage of 2H2OP is the targeted KI of much larger DNA fragments, such as BAC clones, or the replacement of large gene clusters, illustrated in Figs 4 and 5, respectively, without the need to modify the donor vectors. However, the overall efficiency of this BAC KI or cluster gene replacement was low, at 6.7% and 4.3%, respectively. Our injection of the same BAC clone into rat zygotes to produce conventional transgenic animals resulted in a 9.2% efficiency (6/65 pups), where the BAC was randomly integrated (Table 1). Such a low efficiency of BAC transgenesis has been reported by other groups (1–4%)^41^,^42^. The efficiency for the integration or deletion of large regions by 2H2OP may depend on the size of the targeted region as previously reported^43^,^44^.

The disadvantage of 2H2OP is the high rate of indel mutations at ssODN-mediated conjunction sites (Fig. 3d). The lsODN approach could circumvent this problem for precise KI. In the experiment using lsODN targeting the start codon of the *Thy1* locus, many deletion mutations were observed on the 5′-side of the integrated lsODN sequences (Fig. 2d), probably because of the 5′–3′ exonuclease activity of the DSB repair system. These observations provide some clues about the underlying mechanism for repairing CRISPR-mediated DSBs with single-stranded ODNs. We hypothesize that this repair system is the synthesis-dependent strand annealing pathway, which is one of the major HR pathways for repairing DSBs^45^,^46^ (Supplementary Fig. 25). In the synthesis-dependent strand annealing pathway, after resection of the 5′-strand of the DSB, the 3′-strand of the ssODN anneals to the homologous sequences on the 3′-strand of the DSB. Then, DNA synthesis occurs from the 3′-end of the DSB, to copy the remaining 5′-strand of the ssODN. A second annealing occurs with the other 3′-end of the DSB after removal of the ssODN. Finally, gap filling and ligation complete the repair event^18^,^19^,^45^,^46^. The 5′-degradation of ssODNs by exonuclease activity or the removal of the ssODN by helicase activity might concurrently occur with DNA synthesis, which causes incomplete repair, such as indel mutations (Supplementary Fig. 25). In this process, microhomology sequences between the 3′-strand of the DSB and the newly synthesized 3′-strand of the ssODN may also cause indel mutations. In this study, extension of the 5′-side of the homology arms up to 300-bp drastically ameliorated the deletion mutations on the 5′-side of the lsODN sequences, providing the precise 2A-GFP KI at the end of the *Thy1* locus (Fig. 2f,g). The limitation of the lsODN approach is the maximal length of the synthetic lsODN, which is currently limited to ∼3 kb (Nagahora *et al.*, unpublished data).

In this study, we also demonstrated that poly(A) tail elongation enhances the translation efficiency of Cas9 mRNA in zygotes, thereby increasing the efficiency of CRISPR-mediated genome editing (Fig. 1). Poly(A) tails generally play an important role in the stability and translation of most eukaryotic mRNAs^27^,^28^,^29^. In vertebrate oocytes, maternal mRNAs are mostly dormant with relatively short poly(A) tails, usually fewer than 20 nucleotides^27^,^28^,^29^. In contrast, the length of the poly(A) tail is strongly associated with translational efficiency^47^. Interestingly, this association is observed only in oocytes or early-stage embryos, but not in somatic cells or tissues, which suggests that a translational control switch exists in early embryos^47^. It is also reported that attaching synthetic tails or increasing the length of an mRNA increases its translation in *Xenopus*^48^, *Drosophila*^49^ and mouse oocytes^50^,^51^. Consistent with previously published papers^51^, our study confirms that increased levels of Cas9 in embryos produced neither severe toxicity nor off-target effects in other chromosomal regions.

In conclusion, we have established new ssODN-mediated KI approaches with the CRISPR-Cas system, which are applicable to any target site, any species and with any donor vector without the need of attaching homology arms. These technologies facilitate easy and flexible genome engineering in living organisms, such as the production of rainbow animals with colourful reporter genes or cross-species GM (for example, humanized) animals.

## Methods

### Animals

F344/Stm (NBRP-Rat No. 0140) rats were provided by the National Bio Resource Project for the Rat in Japan (www.anim.med.kyoto-u.ac.jp/nbr). Jcl:Wistar rats and C57BL/6JJcl mice were obtained from CLEA Japan Inc. (Tokyo, Japan). The animals were kept under conditions of 50% humidity and a 14:10 h light:dark cycle. The newly developed W-*Rosa*^*em1(CAG-GFP)Kyo*^ rats (NBRP-Rat No. 0770) were deposited into the National Bio Resource Project—Rat in Japan. They were fed a standard pellet diet (F-2, Oriental Yeast Co., Tokyo, Japan) and tap water *ad libitum*. Animal care and experiments conformed to the Guidelines for Animal Experiments of Kyoto University and were approved by the Animal Research Committee of Kyoto University.

### Preparation of Cas9 and gRNA vectors and donor DNA

Plasmid vectors expressing hCas9 (ID#41815) were obtained from the Addgene repository (www.addgene.org/CRISPR) and were modified by addition of the T7 promoter and the SV40 nuclear localization signal at the amino terminus of hCas9, using an In-Fusion HD cloning kit (Takara Bio, Shiga, Japan) according to the manufacturer's protocol. An 81-bp poly(A) was also added to the 3′-untranslated region of hCas9 using the In-Fusion cloning kit. To measure expression intensity in rat embryos, the 2A peptide and an Enhanced GFP cassette (from pCas9-2A-GFP: Addgene ID#44719) was inserted downstream of hCas9. Cas9 mRNA was transcribed *in vitro* using a mMESSAGE mMACHINE T7 Ultra Kit (Life Technologies, Carlsbad, CA, USA) from linearized plasmids and was purified using a MEGAClear kit (Life Technologies).

To design the gRNAs, software tools (crispr.genome-engineering.org) predicting unique target sites throughout the rat genome were used. gRNAs were transcribed *in vitro* using a MEGAshortscript T7 Transcription Kit (Life Technologies) from synthetic double-stranded DNAs that included a T7 promoter, 20-bp target sequences and gRNA tail sequences, obtained from Integrated DNA Technologies (IA, USA). To prepare gRNA, oligonucleotides designed for target sites were also cloned into BsaI-digested pDR274 vectors (Addgene ID#42250). The target sites and sequences of the ssODNs in the rat genome are shown in Supplementary Table 6.

ssODNs were obtained from Integrated DNA Technologies. pCAGGS was obtained from RIKEN BRC, which is participating in the National Bio-Resource Project of the Ministry of Education, Culture, Sports, Science and Technology, Japan. DNA encoding enhanced GFP was inserted into pCAGGS and was verified by sequence analysis. A BAC clone containing the human *SIRPA* gene (RP11-993C19) was obtained from Life Technologies. BAC DNA was purified using NucleoBond BAC 100 (Takara Bio) according to the manufacturer's protocol.

### Long ssODNs

lsODNs were prepared using an LsODN Preparation Kit (Biodynamics Laboratory Inc., Tokyo, Japan) according to the manufacturer's protocol. A DNA fragment comprising a GFP cassette and homology arms was cloned between the BspQI and the BsrDI sites of pLSODN-1. The resulting plasmid was digested with nicking endonucleases Nt.BspQI and Nb.BsrDI. The nicked plasmid was subject to denaturing agarose gel electrophoresis containing urea. ^DynaMarker^Prestain Marker for RNA High conc (Biodynamics Laboratory Inc.) was used as a visible molecular weight marker to monitor electrophoresis in real time. After electrophoresis, the gel was stained with crystal violet solution, which visualized three bands, a single-strand DNA fragment comprising GFP cassette and homology arms, a liner single-strand vector DNA and a single-strand circular whole-plasmid DNA. The band corresponding to a single-strand DNA fragment comprising GFP cassette and homology arms was excised and extracted according to the manufacturer's protocol. LsODNs were aliquoted and stored at −80 °C, to avoid degradation by repetitive freeze–thaw.

### Microinjections into rat and mouse embryos

Rat and mouse females were superovulated by injection with gonadotropin serum from pregnant mares (PMSG: Aska Pharmaceutical Co., Tokyo, Japan) and human chorionic gonadotropin (Aska Pharmaceutical Co.). Next, pronuclear-stage embryos were collected from superovulated rat or mouse females. They were cultured in a modified Krebs–Ringer bicarbonate medium or KSOM medium (ARK Resource, Kumamoto, Japan) before and after microinjections. Using a micromanipulator (Narishige, Tokyo, Japan), 100 ng μl^−1^ Cas9 mRNA, 50 ng μl^−1^ gRNA, 50 μg ml^−1^ ssODNs and 5 μg ml^−1^ plasmids were microinjected into the male pronuclei of embryos. Injected embryos were selected randomly for each experiment. They were cultured in modified Krebs–Ringer bicarbonate or KSOM medium overnight and divided two-cell embryos were transferred into pseudopregnant females.

### Gene expression of KI and mutation detection assay

The expression level of GFP in embryos was measured using a BIOREVO immunofluorescence microscope (Keyence, Osaka, Japan). After incubation for 24 h, two-cell embryos were collected and genomic DNA was amplified using the GenomePlex Single Cell Whole Genome Amplification Kit (Sigma Aldrich, St Louis, MO, USA). After purification of the DNA, CRISPR-Cas-mediated mutations at target sites were analysed by direct sequencing.

For mutation detection, genomic DNA was extracted from tail biopsies using a GENEXTRACTOR TA-100 automatic DNA purification system (Takara Bio). The PCR products amplified with specific primer sets (Supplementary Table 6) were directly sequenced using the BigDye terminator v3.1 cycle sequencing mix and the standard protocol for an Applied Biosystems 3130 DNA Sequencer (Life Technologies).

Total RNA was extracted using Isogen reagent (Nippon Gene) from the spleen of 5-week-old rats. First strand cDNA was synthesized from 5 μg of total RNA that had been treated with DNase using an oligo(dT)12–18 primer and SuperscriptII reverse transcriptase (Invitrogen). PCR was performed with the primers for human *SIRPA* and rat *Sirpa* described in Supplementary Table 6.

### Off-target analysis

Potential off-target sites in the rat genome (rn5) were identified using the latest version of the CRISPR Design Tool website (crispr.mit.edu). All potential sites were ranked by the off-target hit score based on the predicted specificity. High-ranked potential sites were sequenced in the founders to identify any off-target sites (Supplementary Table 2).

### Southern blotting

Genomic DNA was purified from the liver of all founders using phenol–chloroform extraction. After digestion of the DNA with appropriate restriction enzymes, 10-μg samples of digested DNA were separated by electrophoresis on 0.8% agarose gels and then blotted onto positively charged nylon membranes (Roche, Basel, Switzerland). Ultraviolet cross-linked membranes were hybridized with ^32^P-labelled DNA probes (synthesized by random primed labelling), washed and exposed to X-ray film.

### Fluorescent *in situ* hybridization

Spleen cells were cultured in 10 ml of RPMI1640 medium (Sigma) supplemented with fetal bovine serum (20%), concanavalin A (3 μg ml^−1^) and lipopolysaccharide (10 μg ml^−1^) for 68 h. Before harvesting, 5-bromo-2′-deoxyuridine (Sigma) was added for 3.5 h at a concentration of 30 μg ml^−1^ and then colcemid (Sigma) was added for 30 min at a concentration of 0.02 μg ml^−1^. The culture was then treated with hypotonic solution (75 mM KCl) for 20 min and washed three times in fixative (3:1 methanol:acetic acid). After drying overnight, the metaphase spreads were stained with Hoechst 33258 and ultraviolet irradiated for 4 min at 70 °C, to visualize chromosome banding patterns.

The plasmids containing the transgene were labelled by nick translation with digoxigenin-11-dUTP (Roche). The labelled probes were mixed with sonicated salmon sperm DNA and rat cot-1 DNA in hybridization solution. The probe was applied to metaphase spreads, denatured at 70 °C for 5 min and hybridized overnight at 37 °C. The hybridized slide was washed and probe signals were detected with anti-digoxigenin-Cy3 antibody. After mounting with anti-fade mounting solution, FISH images were captured with the CW4000 FISH application programme (Leica Microsystems Imaging Solution, Cambridge, UK) using a cooled charge-coupled device camera mounted on a Leica DMRA2 microscope.

## Additional information

**How to cite this article:** Yoshimi, K. *et al.* ssODN-mediated knock-in with CRISPR-Cas for large genomic regions in zygotes. *Nat. Commun.* 7:10431 doi: 10.1038/ncomms10431 (2016).

## Supplementary Material

Supplementary Information Supplementary Figures 1-25 and Supplementary Tables 1-6

## Figures and Tables

**Table 1:**
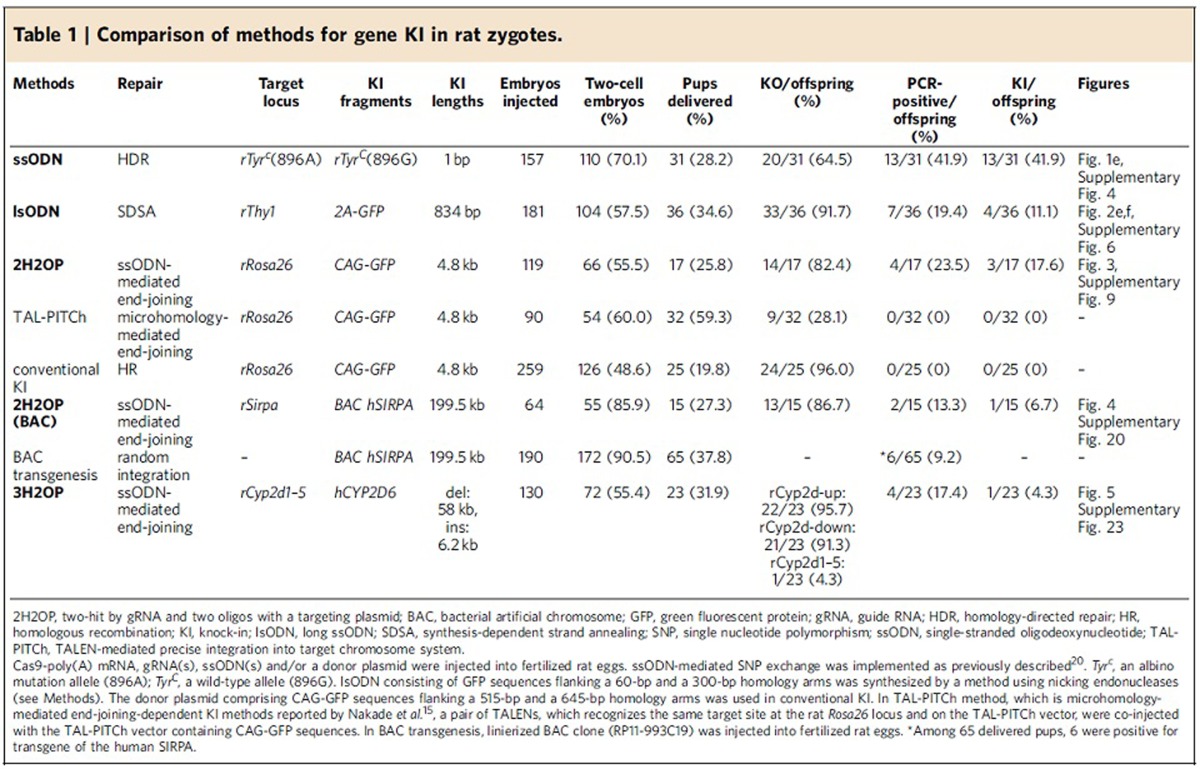
Comparison of methods for gene KI in rat zygotes.
