# Supplementary material for: ssODN-mediated knock-in with CRISPR-Cas for large genomic regions in zygotes
Source: Nat Commun. 2016 Jan 20;7:10431. doi: 10.1038/ncomms10431 (PMC4736110; doi:10.1038/ncomms10431)
Supplement: Supplementary Information — Supplementary Figures 1-25 and Supplementary Tables 1-6 [file ncomms10431-s1.pdf]

CRISPR-mediated KO/KI mutations at rat *Tyr* loci

Wistar	WT		CATGGTTTCCAGGATTATGTAATAGTGGTCCCT
#1	KI/KO*	T>C	CATGGTTTCCAGGATTACGTAATAGTGGTCCCT
		+1	CATGGTTTCCAGGATTATGTAAATAGTGGTCCCT
		-4	CATGGTTTCCAGGATTATGTAA----GGTCCCT
#2	KI/WT	T>C	CATGGTTTCCAGGATTACGTAATAGTGGTCCCT
#3	KI/KO	T>C	CATGGTTTCCAGGATTACGTAATAGTGGTCCCT
		+1	CATGGTTTCCAGGATTATGTAATTAGTGGTCCCT
#4	KI/KO/WT	T>C	CATGGTTTCCAGGATTACGTAATAGTGGTCCCT
		+1	CATGGTTTCCAGGATTATGTAAATAGTGGTCCCT
#5	KI/WT	T>C	CATGGTTTCCAGGATTACGTAATAGTGGTCCCT
#6	KI/WT	T>C	CATGGTTTCCAGGATTACGTAATAGTGGTCCCT
#7	KI/KO	T>C	CATGGTTTCCAGGATTACGTAATAGTGGTCCCT
		+1	CATGGTTTCCAGGATTATGTAAATAGTGGTCCCT
#8	KI/WT	T>C	CATGGTTTCCAGGATTACGTAATAGTGGTCCCT
#9	KI/KO	T>C	CATGGTTTCCAGGATTACGTAATAGTGGTCCCT
		-7	CATGGTTTCCAGGATTA-------GTGGTCCCT
#10	KI/WT	T>C	CATGGTTTCCAGGATTACGTAATAGTGGTCCCT
#11	KI/KO/WT*	T>C	CATGGTTTCCAGGATTACGTAATAGTGGTCCCT
		+1	CATGGTTTCCAGGATTATGTAAATAGTGGTCCCT
#12	KI/WT	T>C	CATGGTTTCCAGGATTACGTAATAGTGGTCCCT
#14	KO	+1	CATGGTTTCCAGGATTATGTAAATAGTGGTCCCT
#15	KO/WT	+1	CATGGTTTCCAGGATTATGTAAATAGTGGTCCCT
#17	KI/KO/WT*	T>C	CATGGTTTCCAGGATTACGTAATAGTGGTCCCT
		-3	CATGGTTTCCAGGATTATGTAAT---GGTCCCT
#18	KO	-1	CATGGTTTCCAGGATTAT-TAATAGTGGTCCCT
		-4	CATGGTTTCCAGGATTATGT----GTGGTCCCT
#19	KO/WT	+1	CATGGTTTCCAGGATTATGTAAATAGTGGTCCCT
#21	KO/WT	+1	CATGGTTTCCAGGATTATGTAAATAGTGGTCCCT
#22	KO/WT	+1	CATGGTTTCCAGGATTATGTAAATAGTGGTCCCT
#24	KO	-3	CATGGTTTCCAGGATTATT---TAGTGGTCCCT
#26	KO/WT	+2	CATGGTTTCCAGGATTATGTAATATAGTGGTCCCT
#27	KO*	+1	CATGGTTTCCAGGATTATGTAAATAGTGGTCCCT
		-3	CATGGTTTCCAGGATTATGTAAT---GGTCCCT
		-6	CATGGTTTCCAGGATTATGT------GGTCCCT
#28	KO/WT	-3	CATGGTTTCCAGGATTATGTAAT---GGTCCCT
#29	KO	+1	CATGGTTTCCAGGATTATGTAATTAGTGGTCCCT
#30	KO/WT	+1	CATGGTTTCCAGGATTATGTAAATAGTGGTCCCT
#31	KO/WT	+1	CATGGTTTCCAGGATTATGTAAATAGTGGTCCCT

Supplementary Figure 1. CRISPR-mediated KO/KI mutation using Cas9-poly(A) plasmids. Cas9-poly(A) mRNA, gRNA targeting of the *Tyr*^{c} allele, and ssODN for recovering the albino phenotype, were microinjected into the male pronucleus of fertilized Wistar rat embryos. Sequence analysis of the 31 pups delivered showed 13 KI alleles, indicated by an orange letter (KI), as well as a variety of indel mutations at the targeted *Tyr* locus, indicated by red letters (KO). Asterisks indicate pups carrying mosaic mutations.

Rat Thy1-ATG EGFP ssODN (+)

TATCACGGGGCTGACATGCAGCTTTCCCCACTGCAGACCCAGGACGGAGCTATTGGCACCATGG
TGAGCAAGGGCGAGGAGCTGTTCACCGGGGTGGTGCCCATCCTGGTCGAGCTGGACGGCGACGT
AAACGGCCACAAGTTCAGCGTGTCCGGCGAGGGCGAGGGCGATGCCACCTACGGCAAGCTGACC
CTGAAGTTCATCTGCACCACCGGCAAGCTGCCCGTGCCCTGGCCCACCCTCGTGACCACCCTGA

EGFP_Small_F
CCTACGGCGTGCAGTGCTTCAGCCGCTACCCCGACCACATGAAGCAGCACGACTTCTTCAAGTC
CGCCATGCCCGAAGGCTACGTCCAGGAGCGCACCATCTTCTTCAAGGACGACGGCAACTACAAG
ACCCGCGCCGAGGTGAAGTTCGAGGGCGACACCCTGGTGAACCGCATCGAGCTGAAGGGCATCG
ACTTCAAGGAGGACGGCAACATCCTGGGGCACAAGCTGGAGTACAACTACAACAGCCACAACGT
EGFP_Small_R

CTATATCATGGCCGACAAGCAGAAGAACGGCATCAAGGTGAACTTCAAGATCCGCCACAACATC
GAGGACGGCAGCGTGCAGCTCGCCGACCACTACCAGCAGAACACCCCCATCGGCGACGGCCCCG
TGCTGCTGCCCGACAACCACTACCTGAGCACCCAGTCCGCCCTGAGCAAAGACCCCAACGAGAA
GCGCGATCACATGGTCCTGCTGGAGTTCGTGACCGCCGCCGGGATCACTCTCGGCATGGACGAG
CTGTACAAGAACCCAGTCATCAGCATCACTCTCCTGCTTTCAGGTACTGGGCAAGGGTCAGGGC
TGGCA

Supplementary Figure 2. Long ssODN (lsODN) used for GFP-KI at the start codon (ATG) of the rat thymus cell antigen 1 (*Thy1*) locus. The lsODN consists of GFP-coding sequences (green) and two 60-bp homology sequences (black) at either end of the GFP. A set of primers used for the PCR analysis is shown in boxes (see also **Supplementary Figure 3**).

Rat *Thy1* locus

*rThy1_ATG*_Small_F
GAGGTGTCCATTGTGTGATCCTTGGATCTCAAGCCCTCACAGTAAATGGGGACCCATCCATCCTATCACGGG

Exon_2 PAM *rThy1_ATG*_target
GCTGACATGCAGCTTTCCCCACTGCAGACCCAGGACGGAGCTATTGGCACCATGAACCCAGTCATCAGCATC

ACTCTCCTGCTTTCAGGTACTGGGCAAGGGTCAGGGCTGGCATTCTAGGGCATCTGGCTTCCTCCCGTCTGG
GGAAGTAGCCTCTTTGCAATAGTCTCAGACACTCGGGTGACTGGGAGTGTGGGGGCGAGCCTCAACCTCACT
*rThy1_ATG*_Small_R

GATCACCTGCTGGTGGTGGTCTTTGACATATTGGAAATTCCATACTGGATCTAAGAACTCCTCTTGCTGGGT
GGTGGTGGTGGTGCACACCTTTAATCCCAGCACTTAGGAGGCAGAGTCAGGTGGGTCTGTAAGTTTGAGGCC
AACCTGGTCTACAGAGTGAGTTCCAGGATATCCAGAACTGTTTGCAAGACAAAGAAACCCTTTCTTGAAAAA
CCATTTAAACACAAACACACACACACACACACACACACACACACATTCTCTCTCTCTCTCTCTCTCTCTCTC
TCTCTCTCTCTCTCTCTCTCTCTCTCTCTCTCTCTCTCCTCTCTTGCTCTCCTGTTCTTGAAACACATTG
TTGGGGCCCAGAACTTCAGTAGATTAGATGGAAATTGAAGGTGGTAGAACATCCCACCAATACCCCAGGGAT
GAGTGCAAAGCTTGAATCCTCCCAGGTCAAGTTTACTTTCCTGCAGGTGGGAGGCCCCTAGTCTGTCTCTCC
CCCAATTCAGAGAAGACACTGCTGTGCCAGTCTTGCAGATGTCCCGAGGACAGAGGGTGATCAGCCTGACAG
CCTGCCTGGTGAACCAGAACCTTCGACTGGACTGCCGTCATGAGAATAACACCAACTTGCCCATCCAGCATG
AGTTCAGCCTGACCCGAGAGAAGAAGAAGCACGTGCTGTCAGGCACCCTGGGGGTTCCCGAGCACACTTACC
GCTCCCGCGTCAACCTTTTCAGTGACCGCTTTATCAAGGTCCTTACTCTAGCCAACTTCACCACCAAGGATG
AGGGCGACTACATGTGTGAACTTCGAGTCTCGGGCCAGAATCCCACAAGCTCCAATAAAACTATCAATGTGA
TCAGAGGTGAGACTGGTCCTCAGAAAAATGGAAGGTCCAGGTTAGCCAGTCCGGGGTAGCCAATGGGCCTGT
AAAACAACAGGCAGCTCCCGATAGCCAGGCCTGAGGGTAGAGGAATGCTCTAGCCATAACTGCTGGTCCCCT
GGGGAACCGCTGCCCTCTGTGTGAGCGGGTGGGGTGGGGGGGATCAAGGGCCAGATAGAGAGAGCTTGCAGT
AAAAAATACAGCTGAGGACACTGCAAATGGGAAAGGTGGTAGCATGGCGGCTCTTGCAGTGTAGAGGTTCTG
TTACACCATCAGGTGGAGCTGCTAAGACATCAAAAAGCACCAGCTCTCTCCCTTGAACAGTCTTTGCCAGCC

*rThy1_TGA*_Small_F Exon_4
TGAGTCTGATCTCCCTGCCTCCCGGTTGGTACCCCTTCTCTCCACAGACAAGCTGGTCAAGTGTGGTGGCAT
AAGCCTGCTGGTTCAAAACACTTCCTGGCTGCTGCTGCTCCTGCTTTCCCTCTCCTTCCTCCAAGCCACGGA

*rThy1_ATG*_target PAM
CTTCATTTCTCTGTGACTGGTTGGGCCCAAGGAGAAACAGGAAACCTCAAGGTCTGCTGAAGAGGTCTTGCT
TCTCCCGGTCAGCTGACTCCCTCCCCAAGACCTTCAAATATCTCAAAACGCGGGGAGAAATGGGGACCCTGT
CCCTCCTAGGGAACCCCAGTGCTGCATGCCATCACCCCCACCCTCACCCCCGCCACTTCACCCTCAGTGCAC
ACCACGAGCTGCCATTTTGTACTCTGTATTCCGGGGCTGCTTCTGATTAGTTTGTCCTGGAGACCCGATGGA
ACACCAGGGTGTATGATGGGCAGTGAAGAGGCAGGATATCACCCCCCCGGGTGAGTTCCTCTTTGCCTTCTA
AGCCAGATGCCTGAAAGAGATATGGATGAGGGAAGTTGGACTGTGTCTGTGCCTGGTATAGTGATACTCTGT
TGAAAGTATCGCCCAGTAGGCA
*rThy1_TGA*_Small_R

Supplementary Figure 3. gRNAs designed to target the start codon (ATG) and the stop codon (TGA) of the rat *Thy1* locus. The gRNA-binding sequences (blue) and the PAM sequences (green) are indicated at the *Thy1* ATG and TGA sequences (red boxes), respectively. Exons are shown as orange boxes. The ssODN sequences are underlined (pink and yellow). The primer sets (small) used for the PCR analysis are shown in boxes (see **Supplementary Table 6**).

CRISPR-mediated KO/KI mutations at rat *Thy1* (ATG) locus

Wistar	WT		AGAGTGATGCTGATGACTGGGTTCATGGTGCCA
#1	KO/WT	+1	AGAGTGATGCTGATGACTGGGTTTCATGGTGCCA
#2	KO/WT*	-7	AGAGTGATGCTGATGACTG-------GGTGCCA
		-8	AGAGTGATGCTGATGACT--------GGTGCCA
#4	KO/WT*	-7	AGAGTGATGCTGATGACTG-------GGTGCCA
		-15	AGAGTGATG---------------ATGGTGCCA
#5	KI/KO/WT	KI-150	AGAGTGATGCTGATGACTGGGTTCTT(GFP)--CCA
		-7	AGAGTGATGCTGATGACTG-------GGTGCCA
#7	KO/WT	-7	AGAGTGATGCTGATGACTG-------GGTGCCA
#8	KO/WT	+1	AGAGTGATGCTGATGACTGGGTTTCATGGTGCCA
#9	KI/WT	KI-171	AGAGTGATGCTGATGACTGGGTTCTT(GFP)---AAG
#10	KO/WT	-7	AGAGTGATGCTGATGACTG-------GGTGCCA
#11	KO/WT	+2	AGAGTGATGCTGATGACTGGGTGTTCATGGTGCCA
#12	KO/WT*	-7	AGAGTGATGCTGATGACTG-------GGTGCCA
		-8	AGAGTGATGCTGATGACT--------GGTGCCA
#14	KO/WT	-7	AGAGTGATGCTGATGACTG-------GGTGCCA
#15	KO	-8	AGAGTGATGCTGATGACT--------GGTGCCA
		-48	TGACC--------(-48bp)--------CCAAT
#16		LD	Large Deletion
#17	KO/WT*	+1	AGAGTGATGCTGATGACTGGGTTTCATGGTGCCA
	KO	-1	AGAGTGATGCTGATGACTGGG-TCATGGTGCCA
#18	KO/WT	-7	AGAGTGATGCTGATGACTG-------GGTGCCA
#19	KO/WT	LD	Large Deletion
#20	KO/WT	+1	AGAGTGATGCTGATGACTGGGTTTCATGGTGCCA
#21	KO/WT*	-9	AGAGTGATGCTGATGAC---------GGTGCCA
		+1	AGAGTGATGCTGATGACTGGGTTTCATGGTGCCA
#22	KO/WT	-11	AGAGTGAT-----------ACACCATGGTGCCA
#23	KO/WT	-7	AGAGTGATGCTGATGACTG-------GGTGCCA
#24	KO/WT	+1	AGAGTGATGCTGATGACTGGGTTTCATGGTGCCA
#25	KO/WT	-7	AGAGTGATGCTGATGACTG-------GGTGCCA
#26	KO/WT	+1	AGAGTGATGCTGATGACTGGGTTTCATGGTGCCA
#27	KO/WT*	-7	AGAGTGATGCTGATGACTG-------GGTGCCA
		-8	AGAGTGATGCTGATGACT--------GGTGCCA
#28	KO/WT*	+1	AGAGTGATGCTGATGACTGGGTTTCATGGTGCCA
		-7	AGAGTGATGCTGATGACTG-------GGTGCCA
#30	KO/WT	-5	AGAGTGATGCTGATGA-----TTCATGGTGCCA
#31	KO/WT	+1	AGAGTGATGCTGATGACTGGGTTTCATGGTGCCA
#33	KO/WT	-7	AGAGTGATGCTGATGACTG-------GGTGCCA

Supplementary Figure 4. NHEJ-mediated KO mutations at the start codon of the rat *Thy1* locus. Cas9-poly(A) mRNA, gRNA targeting the start codon (ATG) of the rat *Thy1* allele, and an lsODN were microinjected into the male pronucleus of fertilized Wistar rat embryos. Sequence analysis of the 33 representative pups delivered showed a variety of indel mutations at the targeted *Thy1* (ATG) locus, as shown by red letters (KO). Asterisks indicate pups carrying mosaic mutations.

Rat Thy1-TGA 2A-EGFP-HA ssODN (-)

CACTGCCCATCATACACCCTGGTGTTCCATCGGGTCTCCAGGACAAACTAATCAGAAGCAGCCC
CGGAATACAGAGTACAAAATGGCAGCTCGTGGTGTGCACTGAGGGTGAAGTGGCGGGGGTGAGG
GTGGGGGTGATGGCATGCAGCACTGGGGTTCCCTAGGAGGGACAGGGTCCCCATTTCTCCCCGC
GTTTTGAGATATTTGAAGGTCTTGGGGAGGGAGTCAGCTGACCGGGAGAAGCAAGACCTCTTCA
GCAGACCTTGAGGTTTCCTGTTTCTCCTTGGCCCAACCAGTCAgttggggtgggcgaagaact
ccagcatgagatccccgcgctgcagTTACTTGTACAGCTCGTCCATGCCGAGAGTGATCCCGGC
GGCGGTCACGAACTCCAGCAGGACCATGTGATCGCGCTTCTCGTTGGGGTCTTTGCTCAGGGCG
GACTGGGTGCTCAGGTAGTGGTTGTCGGGCAGCAGCACGGGGCCGTCGCCGATGGGGGTGTTCT
GCTGGTAGTGGTCGGCGAGCTGCACGCTGCCGTCCTCGATGTTGTGGCGGATCTTGAAGTTCAC
CTTGATGCCGTTCTTCTGCTTGTCGGCCATGATATAGACGTTGTGGCTGTTGTAGTTGTACTCC

EGFP_Small_R
AGCTTGTGCCCCAGGATGTTGCCGTCCTCCTTGAAGTCGATGCCCTTCAGCTCGATGCGGTTCA
CCAGGGTGTCGCCCTCGAACTTCACCTCGGCGCGGGTCTTGTAGTTGCCGTCGTCCTTGAAGAA
GATGGTGCGCTCCTGGACGTAGCCTTCGGGCATGGCGGACTTGAAGAAGTCGTGCTGCTTCATG
TGGTCGGGGTAGCGGCTGAAGCACTGCACGCCGTAGGTCAGGGTGGTC
EGFP_Small_F

ACGAGGGTGGGCCAGGGCACGGGCAGCTTGCCGGTGGTGCAGATGAACTTCAGGGTCAGCTTGC
CGTAGGTGGCATCGCCCTCGCCCTCGCCGGACACGCTGAACTTGTGGCCGTTTACGTCGCCGTC
CAGCTCGACCAGGATGGGCACCACCCCGGTGAACAGCTCCTCGCCCTTGCTCACCATTGGGCCG
GGATTCTCCTCCACGTCACCGCATGTTAGAAGACTTCCTCTGCCCTCTCCGCCGCCGGACCTCA
GAGAAATGAAGTCCGTGGCTTGGAGGAAGGAGAGGGAAAGCAGGAGCAGCAGCAGCCA

Supplementary Figure 5. Long ssODN (lsODN) used for GFP-KI at the stop codon (TGA) of the rat *Thy1* locus. The lsODN consists of GFP-coding sequences (green), 2A peptide sequences (blue), extra 3′-UTR sequences (small), and a 300-bp homology sequence (large) at the 5' end of the lsODN that can be deleted, and a 60-bp homology sequences (black) at the 3' end of the lsODN. A set of primers used for PCR analysis is shown in boxes (see also **Supplementary Figure 3**).

CRISPR-mediated KO/KI mutations at rat *Thy1* (TGA) locus

Wistar	WT		GCCACGGACTTCATTTCTCTGTGACTGGTTGGG
#1	KO	+1	GCCACGGACTTCATTTCTCTGTTGACTGGTTGGG
#2	KO	+1	GCCACGGACTTCATTTCTCTGTTGACTGGTTGGG
#3	KO	+1	GCCACGGACTTCATTTCTCTGTTGACTGGTTGGG
#4	KO	-6	GCCACGGACTTCATTTCTCTG------GTTGGG
		+1	GCCACGGACTTCATTTCTCTGTTGACTGGTTGGG
#5	KO	+1	GCCACGGACTTCATTTCTCTGTTGACTGGTTGGG
#6	KO	+1	GCCACGGACTTCATTTCTCTGTTGACTGGTTGGG
#7	KI/KO	KI	GCCACGGACTTCATTTCTCTG(2A,GFP,3'UTR)TGACT
		+1	GCCACGGACTTCATTTCTCTGTTGACTGGTTGGG
#8	KO	TG>AT	GCCACGGACTTCATTTCTCTGATACTGGTTGGG
		+1	GCCACGGACTTCATTTCTCTGTTGACTGGTTGGG
#9	KI/KO	KI	GCCACGGACTTCATTTCTCTG(2A,GFP,3'UTR)TGACT
		+1	GCCACGGACTTCATTTCTCTGTTGACTGGTTGGG
#10	KI	KI	GCCACGGACTTCATTTCTCTG(2A,GFP,3'UTR)TGACT
#11	KI/KO	KI	GCCACGGACTTCATTTCTCTG(2A,GFP-----
		+1	GCCACGGACTTCATTTCTCTGTTGACTGGTTGGG
#12	KO	-7	GCCACGGACTTCATTTCT-------TGGTTGGG
		+1	GCCACGGACTTCATTTCTCTGTTGACTGGTTGGG
#13	KO	+1	GCCACGGACTTCATTTCTCTGTTGACTGGTTGGG
#14	KO	+1	GCCACGGACTTCATTTCTCTGTTGACTGGTTGGG
#15	KO*	-8	GCCACGGACTTCAT--------GACTGGTTGGG
		+1	GCCACGGACTTCATTTCTCTGTTGACTGGTTGGG
#16	KO	+1	GCCACGGACTTCATTTCTCTGTTGACTGGTTGGG
#17	KO	-1	GCCACGGACTTCATTTCT-GAGAACTGGTTGGG
		+1	GCCACGGACTTCATTTCTCTGTTGACTGGTTGGG
#18	KI/KO*	KI	------------------GFP,3'UTR)TGACT
		-349	GCCACGGACTTCATTTCTCTG---(-349bp)---CCAGA
		-25	GCCA-------------------------TGGG
		+1	GCCACGGACTTCATTTCTCTGTTGACTGGTTGGG
#19	KO	+1	GCCACGGACTTCATTTCTCTGTTGACTGGTTGGG
#20	KO	-10	GCCACGGACTT----------TGACTGGTTGGG
#21	KO	-15	GCCACGGACT---------------GGATTGGG
		+1	GCCACGGACTTCATTTCTCTGTTGACTGGTTGGG
#22	KO	-6	GCCACGGACTTCATTTCTCTG------GTTGGG
#23	KO	-1	GCCACGGACTTCATTTCTCTG-AGCTGGTTGGG
		+1	GCCACGGACTTCATTTCTCTGTTGACTGGTTGGG
#24	KI	KI	GCCACGGACTTCATTTCTCTG(2A,GFP,3'UTR)TGACT
#25	KO	+1	GCCACGGACTTCATTTCTCTGTTGACTGGTTGGG
#26	KO	+1	GCCACGGACTTCATTTCTCTGTTGACTGGTTGGG
#27	KO	+1	GCCACGGACTTCATTTCTCTGTTGACTGGTTGGG
#28	KO	+1	GCCACGGACTTCATTTCTCTGTTGACTGGTTGGG
#30	KO	-6	GCCACGGACTTCATTTCTCTG------GTTGGG
		+1	GCCACGGACTTCATTTCTCTGTTGACTGGTTGGG
#31	KO	+1	GCCACGGACTTCATTTCTCTGTTGACTGGTTGGG
#32	KO/WT	+1	GCCACGGACTTCATTTCTCTGTTGACTGGTTGGG
#33	KO	+1	GCCACGGACTTCATTTCTCTGTTGACTGGTTGGG
#34	KO/WT	+1	GCCACGGACTTCATTTCTCTGTTGACTGGTTGGG
#35	KO	+1	GCCACGGACTTCATTTCTCTGTTGACTGGTTGGG
#36	KO/WT	+1	GCCACGGACTTCATTTCTCTGTTGACTGGTTGGG

Supplementary Figure 6. NHEJ-mediated KO mutations at the stop codon of the rat *Thy1* locus. Cas9-poly(A) mRNA, gRNA targeting the stop codon (TGA) of the rat *Thy1* allele, and an lsODN were microinjected into the male pronucleus of fertilized Wistar rat embryos. Sequence analysis of the 36 pups delivered showed a variety of indel mutations at the targeted *Thy1* (TGA) locus, as shown by red letters (KO). Asterisks indicate pups carrying mosaic mutations.

pCAG-GFP vector (5497bp)

*pCAGGS*_Large_F
TCAAGGCGAGTTACATGATCCCCCATGTTGTGCAAAAAAGCGGTTAGCTCCTTCGGTCCTCCGA
TCGTTGTCAGAAGTAAGTTGGCCGCAGTGTTATCACTCATGGTTATGGCAGCACTGCATAATTC
TCTTACTGTCATGCCATCCGTAAGATGCTTTTCTGTGACTGGTGAGTACTCAACCAAGTCATTC
TGAGAATAGTGTATGCGGCGACCGAGTTGCTCTTGCCCGGCGTCAATACGGGATAATACCGCGC
CACATAGCAGAACTTTAAAAGTGCTCATCATTGGAAAACGTTCTTCGGGGCGAAAACTCTCAAG
GATCTTACCGCTGTTGAGATCCAGTTCGATGTAACCCACTCGTGCACCCAACTGATCTTCAGCA

*pCAGGS*_Small_F
TCTTTTACTTTCACCAGCGTTTCTGGGTGAGCAAAAACAGGAAGGCAAAATGCCGCAAAAAAGG
GAATAAGGGCGACACGGAAATGTTGAATACTCATACTCTTCCTTTTTCAATATTATTGAAGCAT

*pCAGGS*_target PAM
TTATCAGGGTTATTGTCTCATGAGCGGATACATATTTGAATGTATTTAGAAAAATAAACAAATA

*pCAGGS*_Small_R
GGGGTTCCGCGCACATTTCCCCGAAAAGTGCCACCTGGGTCGACATTGATTATTGACTAGTTAT
TAATAGTAATCAATTACGGGGTCATTAGTTCATAGCCCATATATGGAGTTCCGCGTTACATAAC
TTACGGTAAATGGCCCGCCTGGCTGACCGCCCAACGACCCCCGCCCATTGACGTCAATAATGAC
GTATGTTCCCATAGTAACGCCAATAGGGACTTTCCATTGACGTCAATGGGTGGAGTATTTACGG
TAAACTGCCCACTTGGCAGTACATCAAGTGTATCATATGCCAAGTACGCCCCCTATTGACGTCA

*pCAGGS*_Large_R
ATGACGGTAAATGGCCCGCCTGGCATTATGCCCAGTACATGACCTTATGGGACTTTCCTACTTG
GCAGTACATCTACGTATTAGTCATCGCTATTACCATGGTCGAGGTGAGCCCCACGTTCTGCTTC

CAG promoter + 1200bp ⋮

TTCTGGCGTGTGACCGGCGGCTCTAGAGCCTCTGCTAACCATGTTCATGCCTTCTTCTTTTTCC
TACAGCTCCTGGGCAACGTGCTGGTTATTGTGCTGTTAACATGGTGAGCAAGGGCGAGGAGCTG
TTCACCGGGGTGGTGCCCATCCTGGTCGAGCTGGACGGCGACGTAAACGGCCACAAGTTCAGCG
TGTCCGGCGAGGGCGAGGGCGATGCCACCTACGGCAAGCTGACCCTGAAGTTCATCTGCACCAC
CGGCAAGCTGCCCGTGCCCTGGCCCACCCTCGTGACCACCTTCACCTACGGCGTGCAGTGCTTC

GFP protein (726bp)

*GFP*_Small_F
GCCCGCTACCCCGACCACATGAAGCAGCACGACTTCTTCAAGTCCGCCATGCCCGAAGGCTACG
TCCAGGAGCGCACCATCTTCTTCAAGGACGACGGCAACTACAAGACCCGCGCCGAGGTGAAGTT
CGAGGGCGACACCCTGGTGAACCGCATCGAGCTGAAGGGCATCGACTTCAAGGAGGACGGCAAC

*GFP*_Small_R
ATCCTGGGGCACAAGCTGGAGTACAACTACAACAGCCACAAGGTCTATATCACCGCCGACAAGC
AGAAGAACGGCATCAAGGTGAACTTCAAGACCCGCCACAACATCGAGGACGGCAGCGTGCAGCT
CGCCGACCACTACCAGCAGAACACCCCCATCGGCGACGGCCCCGTGCTGCTGCCCGACAACCAC
TACCTGAGCACCCAGTCCGCCCTGAGCAAAGACCCCAACGAGAAGCGCGATCACATGGTCCTGC
TGGAGTTCGTGACCGCCGCCGGGATCACTCTCGGCATGGACGAGCTGTACAAGTAATGATAAAC
TCCTCAGGTGCAGGCTGCCTATCAGAAGGTGGTGGCTGGTGTGGCCAATGCCCTGGCTCACAAA
TACCACTGAGATCTTTTTCCCTCTGCCAAAAATTATGGGGACATCATGAAGCCCCTTG

⋮ **beta-globin poly(A) + 400bp**

Supplementary Figure 7. gRNAs designed to target CAGGS sequences in the CAG-GFP plasmids. The gRNA-binding sequences (blue) and the PAM sequences (green) are shown in the CAGGS sequences. The ssODN sequences are underlined (pink and yellow). The primer sets (small and large) used for the PCR analysis are shown in boxes (see **Supplementary Table 6**).

Rat Rosa26 locus

Exon 1
TCAGAGAGCCTCGGCTAGGTAGGGGATCGGGACTCTGGCGGGAGGGTGGCTTGGCGCGTTTGCG
GGGGCGGGCGGCCGCGGTAGGCCCTCCAAGGACGGTGGAGCCGCTTTGTGGGACAGCTGGGTTC
GATTCGTTAACCCTGGAAGGGGCAAGCGGGTGGTAGTCAGGAATCCGGCCGCCCTGCAGCAACC
GGAGGGGGAGGGAGAAGGGAGCGGAAAAGTCTCCACCGGACGCGGCCATGGCTCCCACGGGGGG
CGGAGAAGCGCTTCCGGTCGATGTCTCATCGCTGATTGGCTGCTTTTCCTCCCGCCGCGTGTGA
AAACACAAATGGCGTGTTTTGGTTGGAGTGAGGCGCCTGTCAATTAACGGCTGCCGGAGTGCGC
AGCCGCTGACTGCCTCGCTGTGCCCACTGGGTGGGGCGGGAGGTAGGTGGGGTGAGGCGAGCTG
GACGTGCGGGCGCGGTCGGCCTCTGGCGGGGCGGGGGAGGGGAGGGTCAGCGAAAGTGGCTGGC
GCGTGAGCGGCCTCCCACCCTCCCCTTCCTCTGGGGGAGTCGTTTTACCCGCCGCCGGCCTGGC

*rRosa26*_Large_F
CTCGTCATCTGATTGGCTCTCGGGGCTCAGAAAACTGGCCTTTGCAATTGGCCCGCGTTCATGC
AAGTTCAGTCCCTAAGCTGGCTGGCGGGGGCGGCAGGGAGGCGCTCACAGGTTCCGGCCCTCCC
CCCAGGCCCCGCGCCGCAGAGTCTGGCCCCGCGCCCCTGCGCAACGTGGCAGGAAGCGCGCGCT
GGGGGCGGGGACGGGCGGTCGGTCTGAGCGGCGGGCGGGTGCAAACGGGATTCCTCCTTGAGTT
GTGGCACTGAGGAACGTGCTGAACAAGACCTACATTGCACTCCAGGGAGTGGATGAAGGAGTTG
GGGCTCAGTCGGGTTGTATTGGAGACAAGAAGCACTTGCTCTCCAAAAGTCGGTTTGAGTTATC

*rRosa26*_Small_F
ATTAAGGGAGCTGCAGTGGAGTAGGCGGAGAAAAGGCCGCACCCTTCTCAGGACGGGGGAGGGG
AGTGTTGCAATACCTTTCTGGGAGTTCTCTGCTGCCTCCTGTCTTCTGAGGACCGCCCTGGGCC

PAM *rRosa26*_target
TGGAAGATTCCCTTCCCCCTTCTTCCCTCGTGATCTGCAACTGGAGTCTTTCTGGAAGATAGGC
GGGAGTCTTCTGGGCAGGCTTAAAGGCTAACCTGGTGCGTGGGGCGTTGTCCTGCAGAGGAATT
GAACAGGTGTAAAATTGGAGGGGCAAGACTTCCCACAGATTTTCGATTGTGTTGTTAAGTATTG

*rRosa26*_Small_R
TAATAGGGGCAAATAAGGGAAATAGACTAGGCACTCACCTGGGGTTTTATGCAGCAAAACTACA
GGTTATTATTGCTTGTGATCCGCCCTGGAGAATTTTTCACCGAGGTAGATTGAAGACATGCCCA
CCCAAATTTTAATATTCTTCCACTTGCGATCCTTGCTACAGTATGAAATTACAGTATCGTGAAT
TAGAATATATAAGCAGAATTTTAAGCATTTTAAAAGAGCCCAGTACTTCATGTCTGTCTCTCCC
ACTTCTGCAGCCCTATCAAAGGGTATTTTAGCACACTCATTTTAGTCCCATTTTCATTTGTTGT
ACTGGCTTATCCAATCCCTAGACAGAGCACTGGCATTCCCTCTCTCCT
*rRosa26*_Large_R

SSupplementary Figure 8. gRNAs designed to target the rat *Rosa26* locus. The gRNA-binding sequences (blue) and the PAM sequences (green) are shown in intron 1 of the rat *Rosa26* sequence. Exon 1 is shown by an orange box. The ssODN sequences are underlined (pink and yellow). The primer sets (small and large) used for the PCR analysis are shown in boxes (see **Supplementary Table 6**).

CRISPR-mediated KO/KI mutations at rat *Rosa26* locus

Wistar	WT		AGAAAGACTCCAGTTGCAGATCACGAGGGAAGA
#1	KO	-1	AGAAAGACTCCAGTTGCAGATCG-CGAGGGAAGA
#2	KO/WT*	-2	AGAAAGACTCCAGTTGCAGA--ACGAGGGAAGA
#3	KO	-6	AGAAAGACTCCAGTTGCA------GAGGGAAGA
		+35	AGAAAGACTCCAGTTGCAGATCATGAG(+35bp)TAATAACGAGGGAAGA
#5	KO/WT*	-2	AGAAAGACTCCAGTTGCAGA--ACGAGGGAAGA
#6	KI/KO/WT*	-6	AGAAAGACTCCAGTTGCAGATCATGAG(pCAG-GFP)CGC------GGGAAGA
		+1	AGAAAGACTCCAGTTGCAGATCGACGAGGGAAGA
#7	KI	+27<br>-256	AGAAAGACTCCAGTTGCAGAGTA(+30bp)GACTGAG(pCAG-GFP)CGCTC<br>(-256bp)CCGCC
#8	KO/Tg?	-506	TACAA-------(-506bp)--------CAGGC
#9	KO	+1	AGAAAGACTCCAGTTGCAGATCAACGAGGGAAGA
		+42	AGAAAGACTCCAGTTGCAGATCATGAG(+46bp)CAATCACGAGGGAAGA
#10	KO/WT*	-106	AGAAAGACTCC-----(-106bp)-----CCCCT
#11	KI/KO	KI	AGAAAGACTCCAGTTGCAGATCATGAG(pCAG-GFP)CGCTCACGAGGGAAGA
		+54	AGAAAGACTCCAGTTGCAGATCATGAG(+54bp)CGCTCACGAGGGAAGA
#12	KO	+11	AGAAAGACTCCAGTTGCAGATCATGAGACAATAACGAGGGAAGA
		+46	AGAAAGACTCCAGTTGCAGATCATGAG(+46bp)AAATAACGAGGGAAGA
#14	KO/WT	+4	AGAAAGACTCCAGTTGCAGATCTGCTACTAGGGAAGA
#15	KO/WT	+5	AGAAAGACTCCAGTTGCAGATCATGAGATGAGGGAAGA
#16	KO	+1	AGAAAGACTCCAGTTGCAGATCACCGAGGGAAGA
#17	KO	-2	AGAAAGACTCCAGTTGCAGA--ACGAGGGAAGA
		+2	AGAAAGACTCCAGTTGCAGATCACACGAGGGAAGA

Supplementary Figure 9. CRISPR-mediated KO/KI mutation at rat *Rosa26* locus. A mix of the Cas9-poly(A), two gRNAs, two ssODNs, and the CAG-GFP plasmids were microinjected into the male pronucleus of fertilized Wistar rat embryos. Sequence analysis of the 17 pups delivered showed three GFP-KI alleles, as indicated by orange letters (KI), as well as a variety of indel mutations at the targeted *Tyr* locus, as shown by red letters (KO). Asterisks indicate pups carrying mosaic mutations.

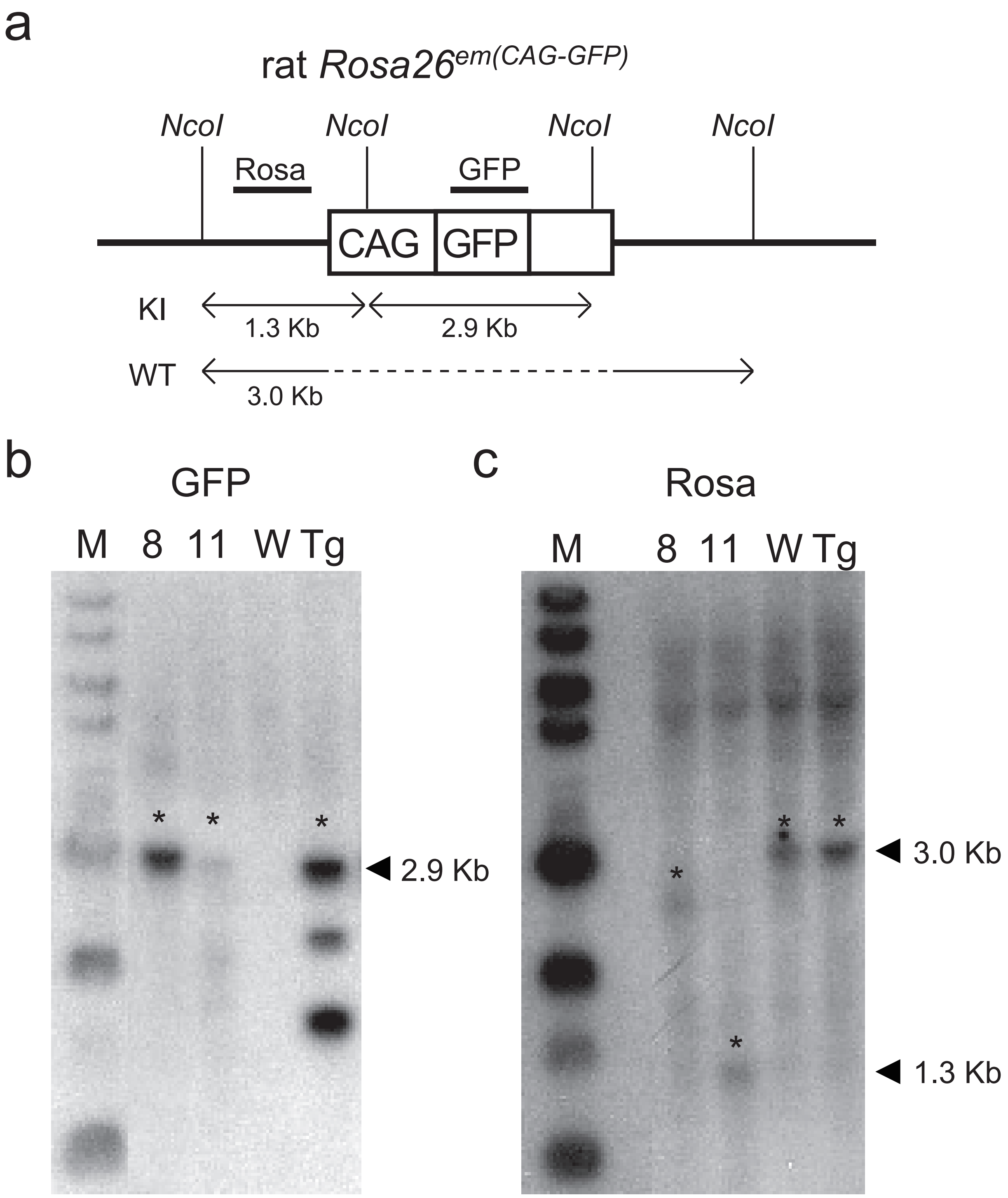

Supplementary Figure 10. Southern blot analysis of the rat *Rosa26* locus in CRISPR-mediated GFP-KI rats. (a) Schematic representation of the rat *Rosa26* and CAG-GFP knock-in loci. *NcoI* restriction sites are indicated. Radioisotope-labeled probes for GFP and Rosa26 were used. Estimated size of each allele, knock-in (KI) or wild-type (WT), is indicated below. (b) The GFP-hybridizing band indicates that rat no. 8 carries three copies of the GFP transgene and no. 11 has one copy. W: Wistar rat as a negative control. Tg: GFP transgenic rat (NBRP-Rat No.0273: W-Tg(CAG-GFP)184Ys) as a positive control. (c) A 1.3-kb short band was detected by *Rosa26* probes in no. 11. A 2.5-kb band corresponding to a 506-bp deletion KO allele was detected in no. 8. The absence of another band detected in no. 8 means that the GFP transgene was randomly inserted at another chromosomal site. Wild-type 3.0-kb bands were detected in the Wistar and GFP transgenic rats.

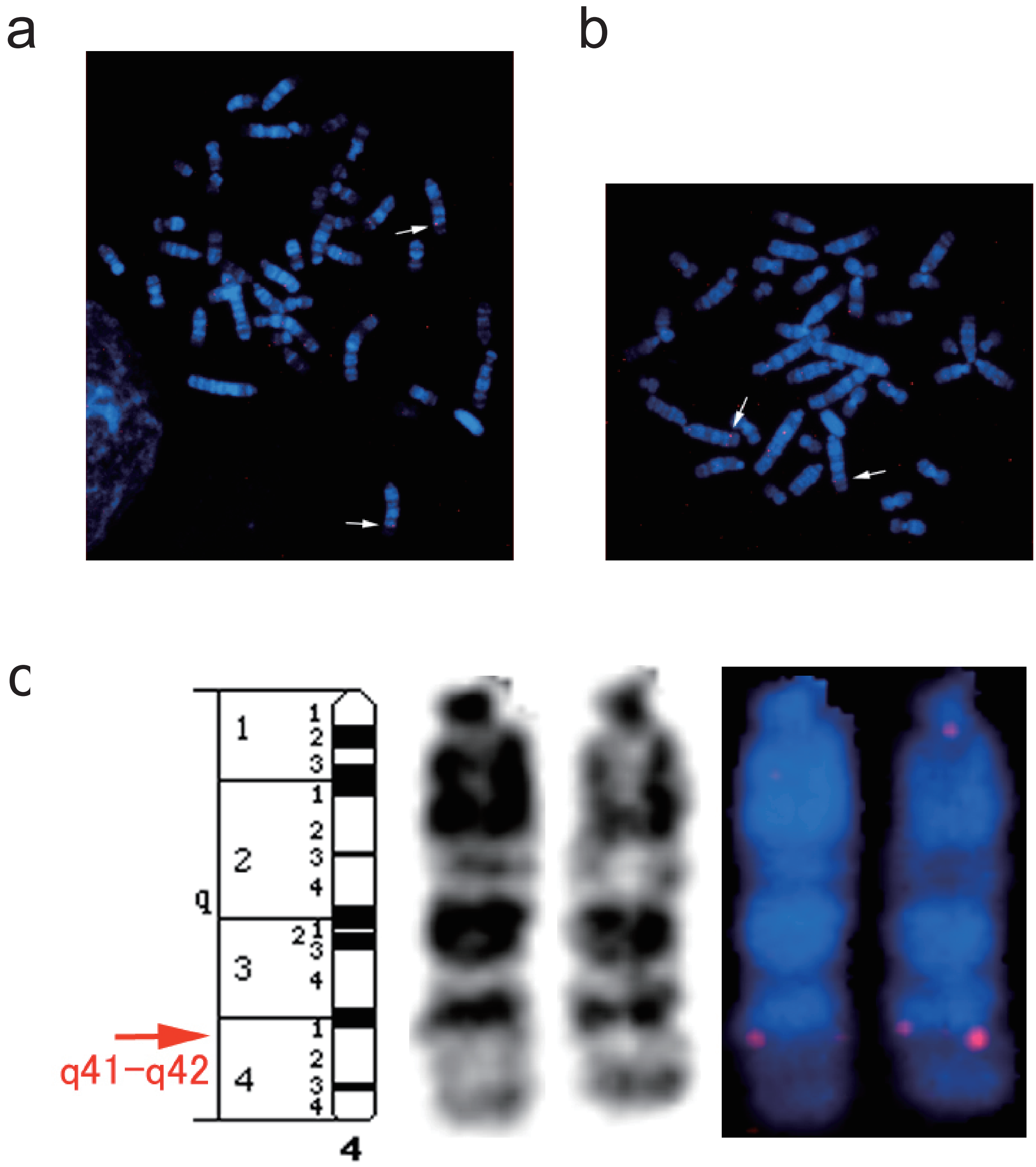

Supplementary Figure 11. Fluorescent in situ hybridization (FISH) analysis of the homozygous GFP-KI rats. The GFP-KI allele hybridized with the fluorescent labeled probes (arrows) (a, b). Homozygous KI alleles were confirmed on rat chromosome 4q41-q42 (c).

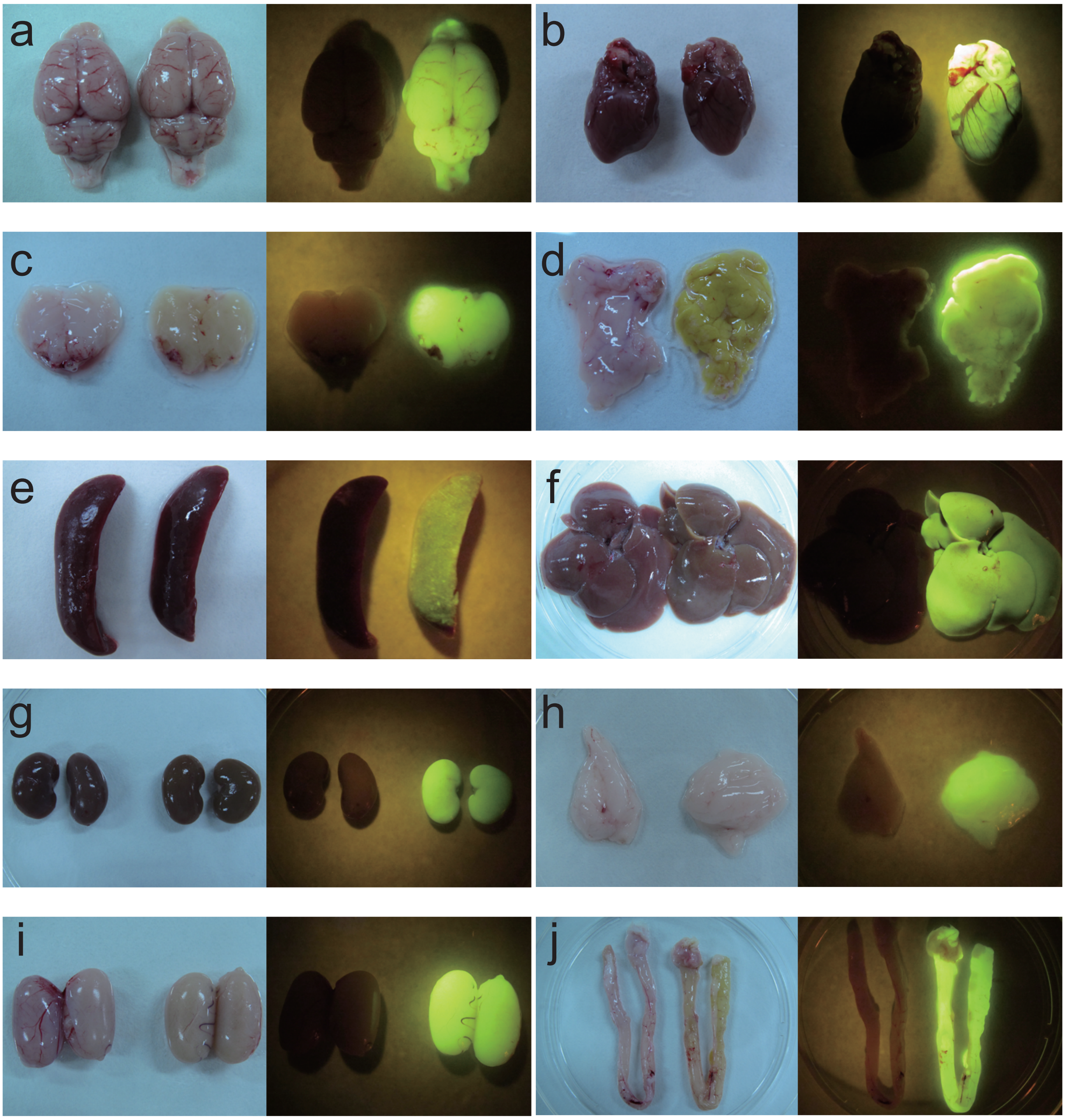

Supplementary Figure 12. Fluorescence photos for GFP-KI rat organs. Each sample was taken from the heterozygous W-*Rosa26*$^{em1(CAG\text{-}GFP)Kyo}$ rats (NBRP-Rat: No.0273) (right) and the control Wistar rat (left). (a) brain, (b) heart, (c) thymus, (d) pancreas, (e) spleen, (f) liver, (g) kidney, (h) adipose tissue, (i) testis, (j) large intestine.

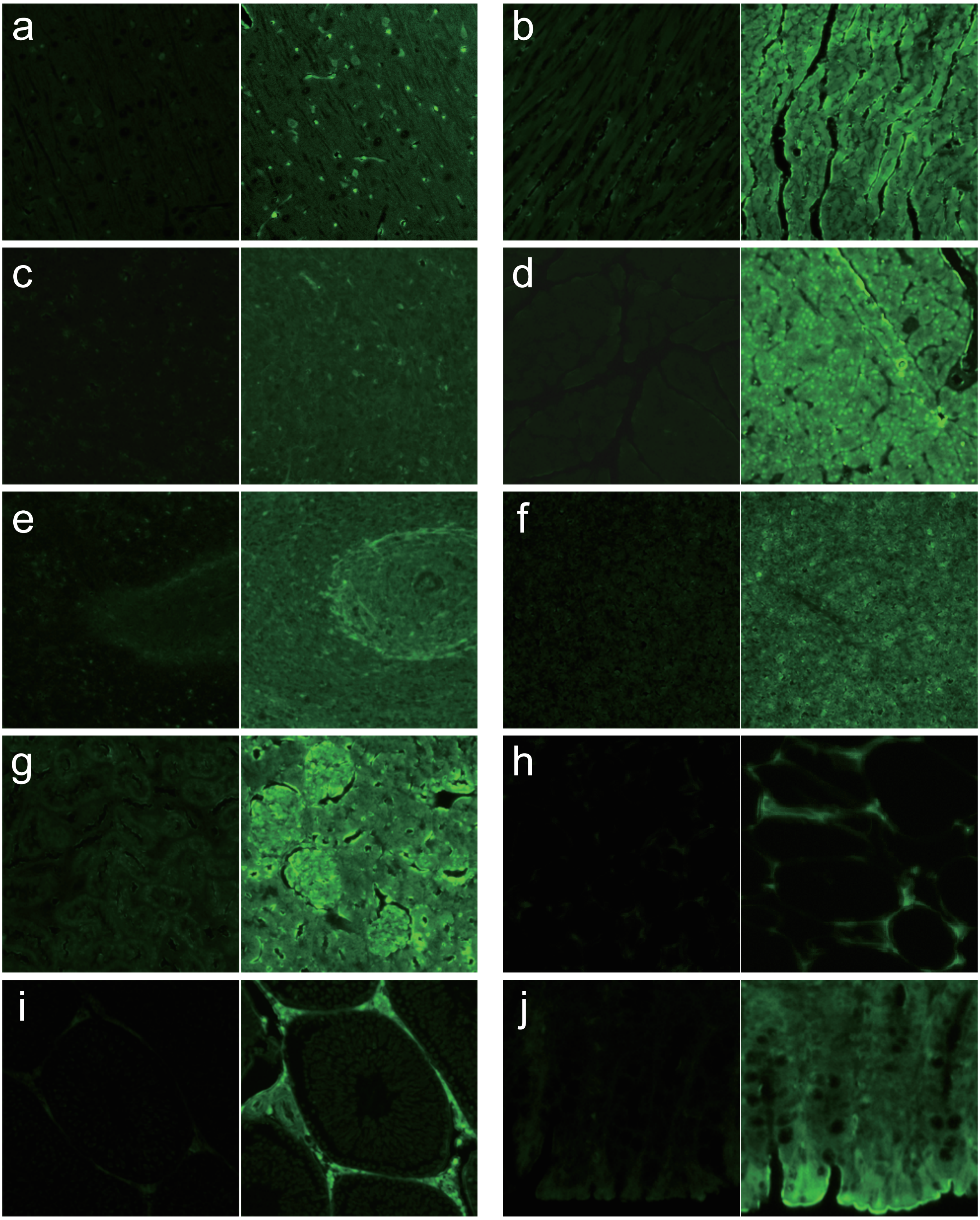

Supplementary Figure 13. Fluorescent tissue sections of GFP-KI rat organs. Each sample was taken from the heterozygous W-*Rosa26*$^{em1(CAG\text{-}GFP)Kyo}$ rats (NBRP-Rat: No.0273) (right) and the control Wistar rat (left). (a) brain, (b) heart, (c) thymus, (d) pancreas, (e) spleen, (f) liver, (g) kidney, (h) adipose tissue, (i) testis, (j) large intestine.

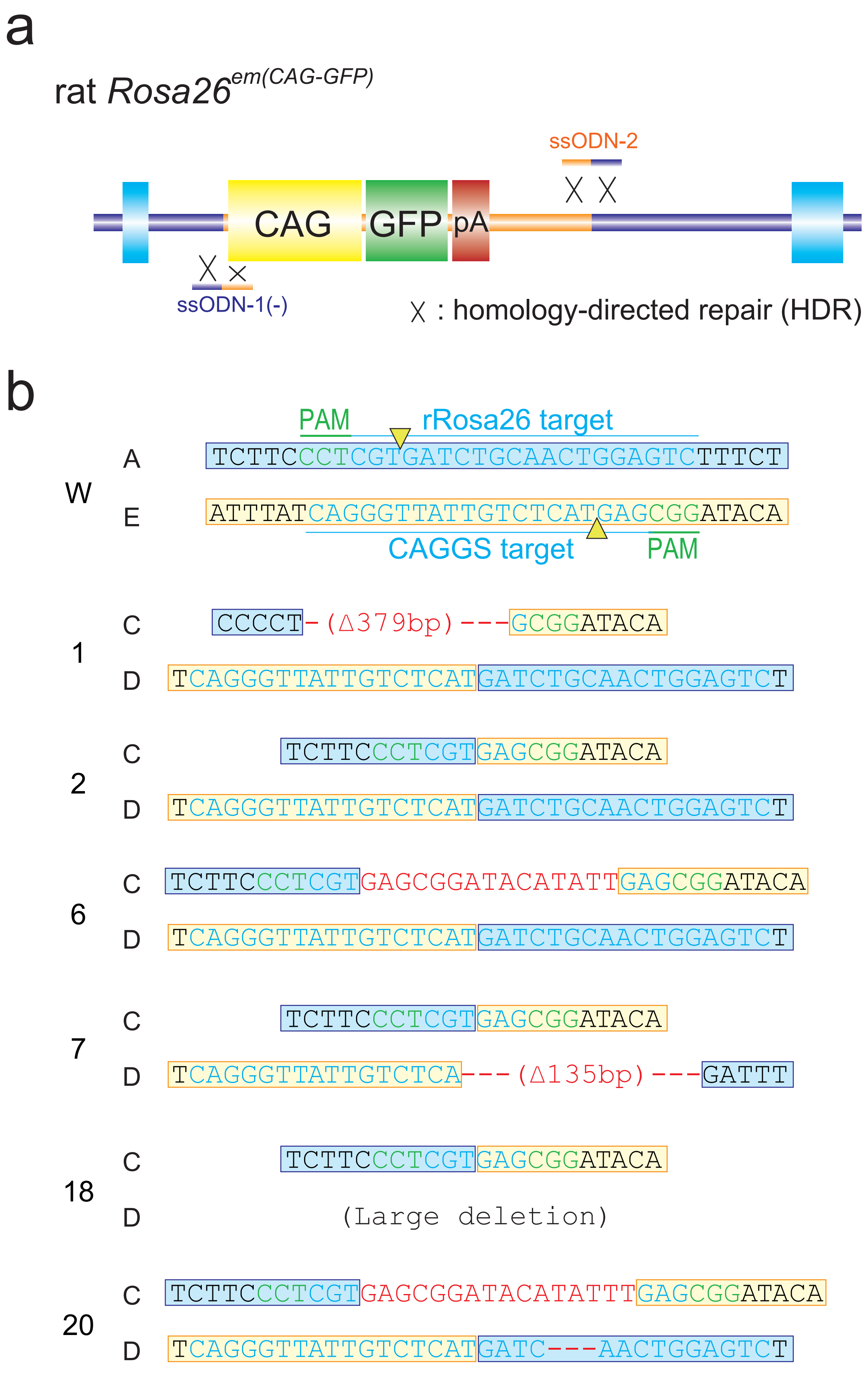

Supplementary Figure 14. CAG-GFP knock-in rats generated by 2H2OP with complementary ssODN (-). (a) Schematic representation of the 2H2OP method with a complementary ssODN-1(-) upstream of the rat *Rosa26* cut site. (b) Sequence analysis of the delivered pups indicated six pups carried GFP-KI alleles.

Mouse Rosa26 locus

Exon_1
TCAGAGAGCCTCGGCTAGGTAGGGGATCGGGACTCTGGCGGGAGGGCGGCTTGGTGCGTTTGCG
GGGATGGGCGGCCGCGGCAGGCCCTCCGAGCGTGGTGGAGCCGTTCTGTGAGACAGCCGGGTAC
GAGTCGTGACGCTGGAAGGGGCAAGCGGGTGGTGGGCAGGAATGCGGTCCGCCCTGCAGCAACC
GGAGGGGGAGGGAGAAGGGAGCGGAAAAGTCTCCACCGGACGCGGCCATGGCTCGGGGGGGGGG
GGGCAGCGGAGGAGCGCTTCCGGCCGACGTCTCGTCGCTGATTGGCTTCTTTTCCTCCCGCCGT
GTGTGAAAACACAAATGGCGTGTTTTGGTTGGCGTAAGGCGCCTGTCAGTTAACGGCAGCCGGA
GTGCGCAGCCGCCGGCAGCCTCGCTCTGCCCACTGGGTGGGGCGGGAGGTAGGTGGGGTGAGGC
GAGCTGGACGTGCGGGCGCGGTCGGCCTCTGGCGGGGCGGGGGAGGGGAGGGAGGGTCAGCGAA
AGTAGCTCGCGCGCGAGCGGCCGCCCACCCTCCCCTTCCTCTGGGGGAGTCGTTTTACCCGCCG

*mRosa26*_Large_F
CCGGCCGGGCCTCGTCGTCTGATTGGCTCTCGGGGCCCAGAAAACTGGCCCTTGCCATTGGCTC
GTGTTCGTGCAAGTTGAGTCCATCCGCCGGCCAGCGGGGGCGGCGAGGAGGCGCTCCCAGGTTC
CGGCCCTCCCCTCGGCCCCGCGCCGCAGAGTCTGGCCGCGCGCCCCTGCGCAACGTGGCAGGAA
GCGCGCGCTGGGGGCGGGGACGGGCAGTAGGGCTGAGCGGCTGCGGGGCGGGTGCAAGCACGTT
TCCGACTTGAGTTGCCTCAAGAGGGGCGTGCTGAGCCAGACCTCCATCGCGCACTCCGGGGAGT
GGAGGGAAGGAGCGAGGGCTCAGTTGGGCTGTTTTGGAGGCAGGAAGCACTTGCTCTCCCAAAG

*mRosa26*_Small_F
TCGCTCTGAGTTGTTATCAGTAAGGGAGCTGCAGTGGAGTAGGCGGGGAGAAGGCCGCACCCTT
CTCCGGAGGGGGGAGGGGAGTGTTGCAATACCTTTCTGGGAGTTCTCTGCTGCCTCCTGGCTTC

PAM *mRosa26*_target
TGAGGACCGCCCTGGGCCTGGGAGAATCCCTTCCCCCTCTTCCCTCGTGATCTGCAACTCCAGT
CTTTCTAGAAGATGGGCGGGAGTCTTCTGGGCAGGCTTAAAGGCTAACCTGGTGTGTGGGCGTT

*mRosa26*_Small_R
GTCCTGCAGGGGAATTGAACAGGTGTAAAATTGGAGGGACAAGACTTCCCACAGATTTTCGGTT
TTGTCGGGAAGTTTTTAATAGGGGCAAATAAGGAAAATGGGAGGATAGGTAGTCATCTGGGGT
TTTATGCAGCAAAACTACAGGTTATTATTGCTTGTGATCCGCCTCGGAGTATTTTCCATCGAGG
TAGATTAAAGACATGCTCACCCGAGTTTTATACTCTCCTGCTTGAGATCCTTACTACAGTATGA
AATTACAGTGTCGCGAGTTAGACTATGTAAGCAGAATTTTAATCATTTTTAAAGAGCCCAGTAC
TTCATATCCATTTCTCCCGCTCCTTCTGCAGCCTTATCAAAAGGTATTTTAGAACACTCATTTT
AGCCCCATTTTCATTTATTATACTGGCTTATCCAACCCCTAGACAGAGCATTGGCATTTTCCCT
TTCCT
*mRosa26*_Large_R

Supplementary Figure 15. gRNAs designed to target mouse *Rosa26* locus. The gRNA-binding sequences (blue) and the PAM sequences (green) are shown in intron 1 of the mouse *Rosa26* sequence. Exon 1 is shown in an orange box. The ssODN sequences are underlined (pink and yellow). The primer sets (small and large) used for the PCR analysis are shown in boxes (see **Supplementary Table 6**).

CRISPR-mediated KO/KI mutations at mouse *Rosa26* locus

C57BL/6	WT		TCTAGAAAGACTGGAGTTGCAGATCACGAGGGAAGAGGGG
#1-1	KI/KO	KI	AGACTGGAGTTGCAGATCATGAG(pCAG-GFP)CGCTCACGAGGGAAG
		+1	TCTAGAAAGACTGGAGTTGCAGATCGACGAGGGAAGAGGGG
#1-2	KO	+29	AGACTGGAGTTGCAGATCATGAG(+29bp)CTTCAACGAGGGAAG
		+10	TCTAGAAAGACTGGAGTTGCAGATCATGCGACATTGGGAGGGAAG
#1-3	KO	+1	TCTAGAAAGACTGGAGTTGCAGATCCACGAGGGAAGAGGGG
		-6	TCTAGAAAGACTGGAGTTGCA------GAGGGAAGAGGGG
#1-4	KO	+1	TCTAGAAAGACTGGAGTTGCAGATCGACGAGGGAAGAGGGG
#1-5	KO	-18	TCTAGAAAGACT------------------GGAAGAGGGG
#1-6	KO	-60	GTTAGAGG-------(-63bp)-------GGGAAGAGGGG

Supplementary Figure 16. CRISPR-mediated KO/KI mutation at the mouse *Rosa26* locus. A mix of Cas9-poly(A), two gRNAs, two ssODNs, and the CAG-GFP plasmid were microinjected into the male pronucleus of fertilized C57BL/6J mouse embryos. Sequence analysis of the representative six pups delivered showed GFP-KI alleles (#1-1), as shown by orange letters, as well as a variety of indel mutations at the targeted *Rosa26* locus, as shown by red letters (KO).

a

Embryos injected	Two-cell embryos (%)	Pups delivered (%)	Knockout (%)	Knock-in (%)
165	132 (80.0)	31 (23.5)	31/31 (100)	3/31 (9.7)

b

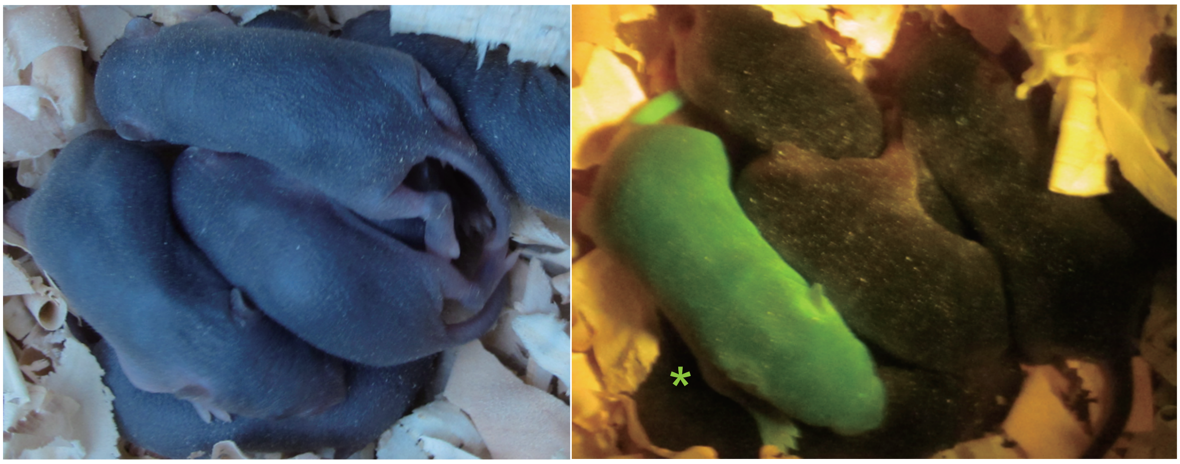

c

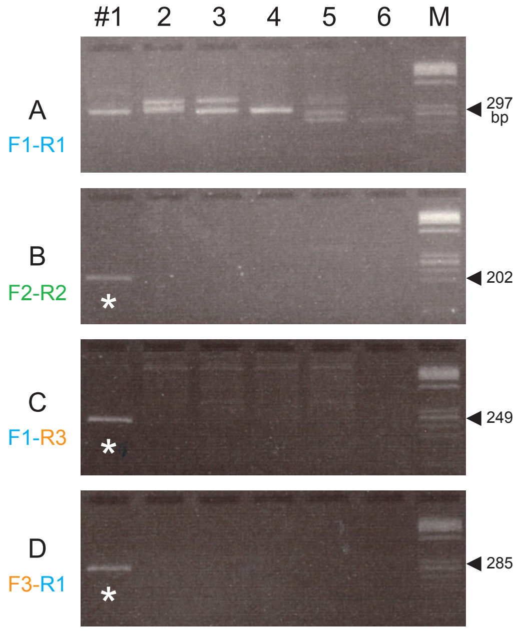

d

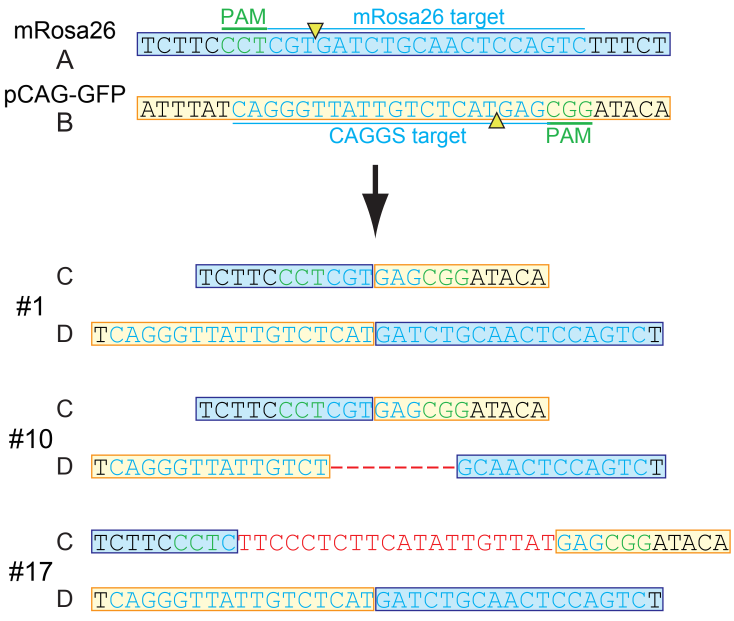

Supplementary Figure 17. CAG-GFP KI mice generated by two-hit gRNA and two-oligo with a targeting plasmid (2H2OP). (a) Cas9-poly(A) mRNA, two gRNAs, two ssODNs, and the CAG-GFP plasmids were co-injected into C57BL/6J mouse embryos. (b) Photos of pups delivered from C57BL/6J mouse embryos injected with a mix of Cas9 mRNA, two gRNAs, two ssODNs, and CAG-GFP plasmid (left). One of the pups expressing GFP at high levels in the body (asterisk) (right). (c) PCR analysis on the pups with primer sets, F1–3 and R1–3, as indicated in **Figure 3**. Three pups showed a GFP positive PCR band (asterisk). No. 1 was positive for the ligation either side of *Rosa26* and the CAG-GFP (C and D). (d) Sequence analysis of the pups indicated that No 1 carried the accurate conjunction at both cut ends.

Rat Sirpa Exon2

*rSirpa*_Large_F

TTCAGGCTCCCATTTTTGTCTCAGGGTTTCTATTCCTGCACAAAACATCATGACCAAGAAGTAA
GTTGGGGAGGGAAGGGTTTATTCAGCTTACACTTCCACATTACTGTTCATCACCAAAGGAAGTC
AGGACTGGAACTCACACAGGGCAGGAAGCAGGAGCTGATGCAGAGGCCACGGAGGGTGCTGCTT
CCTGGATGGCTTCCCCTGGCTTGCTCAGCCTGCTCTCTTATAGAACCCAGTACTACCAGCCCAG
GGATGGCACCACCCATGATCAGTAATTGAGAAAATGCCTTACAGATGGGTCTCATGGAGGCCTT
TCCTCAAGGGAGGCGCCTTTCTCTGTATTAACTACAGCTGTGTCAAGTTGATACACAAAACCAG
CCAGTACACATTTTCACTGACAAATTCAGATTTGAATTTCAGAGCCCAGCTCAGATAGGACATT
TCCCCCCAAAGGGCTTGGGCACTCTACTACACCAAACCATGTGACTTAGCCCTCTGTGCCCCAG
TTTTCTCACCACAGTCTTGATTTACAGGTTCATGTGGGGGATAACACATGCCGAACATTGTGCT
GAACTCAATACCACTGTGTGTGATCAATCAGTGAAAACCATTGATAAACTTGAGCTTGTAGGAT

*rSirpa*_Small_F Exon_2

CCCTTAAGGTGAATCATGGCTGCCATCTTTCTCTCCAGGAGCCAGCGGGAAAGAACTGAAGGTG
ACTCAGGCTGACAAATCAGTGTCTGTTGCTGCTGGAGATTCGGCCACTCTGAACTGCACTGTGT

*rSirpa*_target PAM

CCTCCCTGACGCCTGTGGGACCCATTAAGTGGTTCAAAGGAGAAGGGCAAAATCGGAGCCCGAT
CTACAGTTTCATAGGAGGAGAACACTTTCCTCGAATTACAAATGTTTCAGATGCTACTAAGAGA

*rSirpa*_Small_R

AACAATATGGACTTTAGCATCTGTATCAGTAATGTCACCCCAGAAGATGCTGGCACCTACTACT
GTGTGAAGTTCCAGAAAGGAATAGTAGAGCCTGACACAGAAATTAAATCTGGAGGGGGAACAAC
GCTCTATGTACTCGGTAAGTAGGCTGTGCCTTCCTCATCCCCTCAGGTGTAGCATTAGGTCAGA
GAGTAGCATCCCGGTGAGTGTGGCTCACAAGGGCACAATCTGATCTCAACCTTCAGCAGGTCAG
GGACGTCAAGGCCAATCGTTAGCTCCAGGTTCCAGTGCTAGCAAGTAAGAGGGCATTAGCATCC
CTCATCTGCCCGCCCCACGGCCCTCTCTTCCTTGCACACGGTTCAAGTACCATGAGTACCAGAA
TGCAGATCCCAGCAGGCTCCTTGCTGCCCAGGTATTAGGCATCCCCACAGGTGAGCCCAGGACC
TAGGAAGCACTGAACCTCTAGGAGATGCAGCCTCTCCACACATCACTTTCACAGAGATTCAGGA
AGCCCCTTCCATTCAGAACCCGAGGCCTGTCAGTGACAGAAAGCTCTCCCACATGTCAGTCAGG
ACACAAAGGACCCCTCCATTATATAAATGGCTCCATTTTACTCTAGCATCTAGAAGTTCCACAA
TTTATTGTAGTCTGTTGGTAGATAATTCAGTTTTTCCATGCTGTGTATTTTTTTAAAATAATGC
TGCATCGAATGCC

*rSirpa*_Large_R

Supplementary Figure 18. gRNAs designed to target the rat *Sirpa* locus. The gRNA-binding sequences (blue) and the PAM sequences (green) are shown in exon 2 of the rat *Sirpa* sequence (orange box). The ssODN sequences are underlined (navy). The primer sets (small and large) used for the PCR analysis are shown in boxes (see **Supplementary Table 6**).

pBACe3.6-PISecI site

```
PISecI_Large_F
TCCGGCCTTTATTCACATTCTTGCCCGCCTGATGAATGCTCATCCGGAGTTCCGTATGGCAATG
AAAGACGGTGAGCTGGTGATATGGGATAGTGTTCACCCTTGTTACACCGTTTTCCATGAGCAAA
CTGAAACGTTTTCATCGCTCTGGAGTGAATACCACGACGATTTCCGGCAGTTTCTACACATATA
TTCGCAAGATGTGGCGTGTTACGGTGAAAACCTGGCCTATTTCCCTAAAGGGTTTATTGAGAAT
ATGTTTTTCGTCTCAGCCAATCCCTGGGTGAGTTTCACCAGTTTTGATTTAAACGTGGCCAATA
TGGACAACTTCTTCGCCCCCGTTTTCACCATGGGCAAATATTATACGCAAGGCGACAAGGTGCT
GATGCCGCTGGCGATTCAGGTTCATCATGCCGTTTGTGATGGCTTCCATGTCGGCAGAATGCTT
AATGAATTACAACAGTACTGCGATGAGTGGCAGGGCGGGGCGTAATTTTTTTAAGGCAGTTATT
PISecI_Small_F
GGTGCCCTTAAACGCCTGGTTGCTACGCCTGAATAAGTGATAATAAGCGGATGAATGGCAGAAA
TTCGATGATAAGCTGTCAAACATGAGAATTGGTCGACGGCGCGCCAAAGCTTGCATGCCTGCAG
CCGCGTAACCTGGCAAAATCGGTTACGGTTGAGTAATAAATGGATGCCCTGCGTAAGCGGGGCA
 PI-SceI   PAM     PISecI_target                          LoxP
CATTTCATTACCTCTTTCTCCGCACCCGACATAGATAATAACTTCGTATAGTATACATTATACG
AAGTTATCTAGTAGACTTAATTAAGGATCGATCCGGCGCGCCAATAGTCATGCCCCGCGCCCAC
CGGAAGGAGCTGACTGGGTTGAAGGCTCTCAAGGGCATCGGTCGAGCTTGACATTGTAGGACTA
           PISecI_Small_R
                                         T7
TATTGCTCTAATAAATTTGCGGCCGCTAATACGACTCACTATAGGGAGAGGATCCGCGGAATTC
GAGCTCACGCGTACTGATGCATGATCCGGGTTTAAACCCAGTACTCTAGATCCTCTAGAGTCGA
CCTGCAGGCATGCAAGCTTGGCGTAATCATGGTCATAGCTGTTTCCTGTGTGAAATTGTTATCC
GCTCACAATTCCACACAACATACGAGCCGGAAGCATAAAGTGTAAAGCCTGGGGTGCCTAATGA
GTGAGCTAACTCACATTAATTGCGTTGCGCTCACTGCCCGCTTTCCAGTCGGGAAACCTGTCGT
GCCAGCTGCATTAATGAATCGGCCAACGCGCGGGGAGAGGCGGTTTGCGTATTGGGCGCTCTTC
CGCTTCCTCGCTCACTGACTCGCTGCGCTCGGTCGTTCGGCTGCGGCGAGCGGTATCAGCTCAC
TCAAAGGCGGTAATACGGTTATCCACAGAATCAGGGGATAACGCAGGAAAGAACATGTGAGCAA
AAGGCCAGCAAAAGGCCAGGAACCGTAAAAAGGCCGCGTTGCTGGCGTTTTTCCATAGGCTCCG
CCCCCCTGACGAGCATCACAAAAATCGACGCTCAAGTCAGAGGTGGCGAAACCCGACAGGACTA
TAAAGATACCAGGCGTTTCCC
         PISecI_Large_R
```

Supplementary Figure 19. gRNAs designed to target PI-SceI sequences in the human BAC plasmid. The gRNA-binding sequences (blue) and the PAM sequences (green) are shown in the PI-SceI sequence (yellow). The two ssODN sequences are underlined (pink and yellow). The primer sets (small and large) used for the PCR analysis are shown in boxes (see **Supplementary Table 6**).

CRISPR-mediated KO/KI mutations at rat *Sirpa* locus

Wistar	WT		GCACTGTGTCCTCCCTGACGCCTGTGGGACCCA
#1	KO	+93	GCACTGTGTCCTCCCTGACGCCAAGAG(+93bp)GGAGATGTGGGACCCA
#2	KI/KO	KI	GCACTGTGTCCTCCCTGACGCCAAGAG(BAC-hSIRPA)GGAGATGTGGGACCCA
		+72	GCACTGTGTCCTCCCTGACGCCAAGAG(+72bp)GGAGATGTGGGACCCATTAA
#3	KO	+1	GCACTGTGTCCTCCCTGACGCCTTGTGGGACCCATTAA
#4	KO	+10	GCACTGTGTCCTCCCT------ATGTC(+16bp)GGAGATGTGGGACCCATTAA
#5	KO	+88	GCACTGTGTCCTCCCTGACGCCAAGAG(+88bp)GGAGATGTGGGACCCATTAA
#6	KO	-83	GCACTGTGTCCTC-----(-83bp)-----ACTTT
#7	KO*	-91	GCACTGTGTCCTCCCTGACGC-----(-91bp)-----ACTAA
#8	KO/WT	-2	GCACTGTGTCCTCCCTGACG--TCAGGGACCCA
#10	KO	-11	GCACTGTGTCCTCCCTGAC-----------CCATT
		-93	GCACTGTGTCCTCCCTGAC---(-93bp)---AAATG
#11	KO/WT*	-10	GCACTGTGTCCTCCCTGAC----------CCCAT
#12	KO	LD	Large Deletion
#14	KO	+1	GCACTGTGTCCTCCCTGACGCCGTGTGGGACCCA
#15	KO/WT*	+89	GCACTGTGTCCTCCCTGACGCCAAGAG(+89bp)GGAGATGTGGGACCCA

Supplementary Figure 20. NHEJ-mediated KO mutations at the rat *Sirpa* locus. Cas9-poly(A) mRNA and gRNA targeting exon 2 of the rat *Sirpa* allele were microinjected into the male pronucleus of fertilized Wistar rat embryos. Sequence analysis of the seven pups delivered showed a variety of indel mutations at the targeted *Sirpa* locus, as shown by red letters (KO). Asterisks indicate pups carrying mosaic mutations.

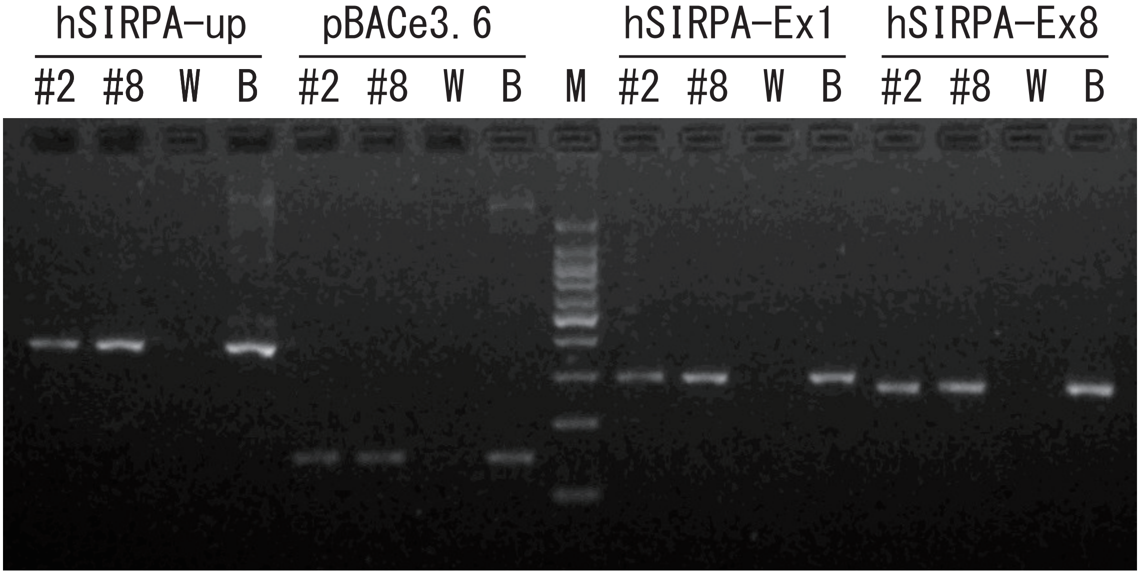

Supplementary Figure 21. PCR analysis for the SIRPA BAC knock-in. Primer sets amplifying the upstream region (hSIRPA-up), exon 1 (hSIRPA-Ex1), and exon 8 (hSIRPA-Ex8) of the human SIRPA gene, and the BAC vector sequences (pBACe3.6) were used (see **Supplementary Table 6**). BAC KI rat no. 2 (#2), BAC Tg rat no. 8 (#8), Wistar control rat (W), human BAC clone, RP11-933C19 (B).

rCyp2d1-5 (58Kbp)

```
rCyp2d-up_Large_F
GACCTCCCACACCTGTCTGTGTACTTATCTCACAGGACTGAGCTGGCTAGGTTGTCCCCAGGCCTGCAAAGAGGC
TGGGGCCCATCTGGTTCTCAGGCTCTTGGGCATCCAGGCACCACTGGAAACTGAAAGAGCTGAGAACAGAGTGGA
GGCCCGCAGGCTGGATCTAGCCGGAGGCTGAGTGGGAAAGGGGTGCTCCAGCTGGTCCTGCAGGGGGAGTCCTTG
GCTCAATGGTAGCAGGTTTCACTTAGCTTGGTGGTGGCTGCCTCTGGGAGCAAAGTGAGGCCTTCTATGGCCTTC
TCTGTGGCTTTCTGTGGCAGATCCCAATGAAAAGAGACAGTCCATGATTTTAAGGTATTTATTCTCATGGTAGAA
AGTGGATGAGTAGAACTGTACTGCACTTCTGAGGGTGGGCCTCAGATTAAGTACTTTTGCAGGAATGAGTGTTTG
GGAAGAAAGGTTATTGGCTCAGTCTCCAGGCTTTTAGGTACCTCATTATAATGGAGATCTGACTTGAAATGACCA
GGTCACCTCCTCTCCATGTGGAGGGGCTTGGGGCATTGCCCTTACATGACTGATGGCAACAAATGTATGGGGGGC
rCyp2d-up_Small_F
                                                 rCyp2d-up_target PAM
TGCGGCTAGTGACAGGGCCTGGTGCCCAGGAGTCAGGCAAAACACCTACCGTCTCTTCAGGGTAACTGTGGTTTT
AAGGCTTAGCTCAACTGGGAATCAGGGTGCCTTTGACAGTCCCACAGGGATTGACCTATCAAGAAGACAAGGATG
AATGAAATTAGATAGTGGTCTCCATGAGACAAGCACCTGGACTCCTAGCTGGTCTCCCCAGGACTCTTGACAGTT
                               rCyp2d-up_Small_R
GGTGACAACCTTTCAGCTTTGTCAGGTGAGAGCTAAGAAGTCCCTAGGTTTTGAATTTGGCCATCCTTGAGACTT
GAACGACCTTCCACCTGTACTTGAAGCTTCACAGGCTGGAGTTACCTTCCTCACACTGCCTCTCCCTCACCCTCA
TCAGCTACCCACACAAGCCACTTCAGTGGCCCTTGCTGCATACTGATAGTGGCCATGGTGTCAGTCAGACCCCAT
GTCTGACTAGCCAAGTGTTTCTGGAAGCCTCCAAAAGTTCCTCTTCCAATCTGAGACGATGCCCTCAGGCCCCGT
GGATCTCTGCCCATAAGGCAAATCAGCAGCTTGCACACTGAGGAAGGGTTCATGTTATGTGCTTGTCTTGCTCAA
AATGTCCAGCCCAACATAACCACTATGCTGCTTTCTGTGGAGATTTCAATGCCACTCCCATGGACAAGCACCACC
                                                         rCyp2d-up_Large_R
GGAGAGAAGAAAGGGCAAGAACCTCTGATGTTTATTTTCCCTGGACTTTTCTGACACCCCTTCCCCCATACAGGC
TCTGTTTGAACATTAACACAGCTACTTGGCTCAATTTAGAGAACAAAGTGCAGCTTTACCCCCTTTTAAGGGACC
AGGTTCCCAGTCTGTTGTAAGAGGATCTTTCCTGATTCTAAGTGTCTGGACAAGTATTCCTTCAAAAGCCTGCAA
GCCGTGGGGCAGCCATGGGGCTGCTGATTGGAGATGACCTCTGGGCTGTGGTCATATTCACAGCCATCTTCTTGC
```

rCyp2d1-5 55Kbp

```
ACTATGGCGTCTTTGGTGCTCTGACCACCCCGCGCCCCTACCAGCTCTGTGCTTCACCCCGCTAAGGGGAGGCAC
AGCATCTCACTCACTGTGCTTGCTGGGGTCCTAGTGTGCAATAAATGGTTTTACTCTGAACCGAATCATCCCTGT
GAGCTCTCCAGGCTGTAAGGGGCCTGAGCAGCCTTCCCGTGGACATCCGCACCCCTACTTAATCTTCCTTGACCA
TGTGCCCCAATGGAAGGGCTGCTCTACTGACCTCCGAAATGGCAGCCATTCTTGCTTTCACCCCTGCCCCCTCTT
                               rCyp2d-down_Large_F
TTCACCCAAATTGATGATGTTTATTCATAGATGCCAACATCTGGAAGGAGGGCCAGAAAGGACTGCTGTGAAGGG
TCAGTGTAAGTCACACAGATGAGGGAAGGGGCGGTGGAGGTAATGGTGGGCAGAATTGTCCCCTTTCCACTTGAG
ATGTTTCTCCCAGACGCCCCCATTTCAGACCCACTACACAACCAAGGCTAACTCCTCAGCCAGCATCATCACAAC
TTCTTATATGACGTCGCAGAGATGTAGAGAAGTCGGGGAGGCTGGAAATGACATGCAGGTTAAGTGCCCAAGGTT
ACCTGTTGGGTACCACATGCTTCCCTAAACGGTTTTGTGGGGGTCCAGAAGCAGGTTGCCTCCTAAGCTTCTTTG
TCACCATTAATTCCATGACCCAGCAGGGATACTGGTGTCCAGGCCCATGCACAGTAAGAAAGTGACTCTAACCAG
GGATGGAAGGACCCGCAAGCTTAGTGTTGACACAGACTCCCAGACCTTAGCACAACTGACTCCATGGTAGAAGTA
CCATTTGGGCCATAAAACTTAGCACGTAGACAGCAGCTCCTCTCATAATGAAAACAAAGACCTAACCCATCAAAT
       PAM        rCyp2d-down_Small_F                        rCyp2d-down_target
TCTATCCTGGGAAGGTCTCTTGAAGCACTCCTCTTGGCTTCTTGGCTTCTGTAGTTCTCCTAGCTAACTGCTCTT
GCTAACTGAAGTATGTCAACCCAGGATATGGTTGTTGGTAAAAGCTCGCCCTGAGAACAGCTCAGGACGACATTG
AGGTGACCCAGTGTAGTCACCAGCCAGCTAATAAAGACCTCCTTTTGGTTTAAATCCATATCTGAGTAGTCTTCT
      rCyp2d-down_Small_R
CTGGTGCATACCTCACACCATTTCTAAAGGTTGCAACAAGATCCCTAGAGACAGACCTTGAGGCACCATGGGTCT
CAGATCCCCATGGTGCAGAGAAGAGGAGTATGGTAGTCTAGGGGCTCCCAGGAAGTGTGCAACCAGAAGACTTTC
CAGGGCCTTAGGACTGCCTTTGATCATTTGCTGCCTAAAAGCTTTCTGACACTGCACCTCCCCCCCCAAAAGAAA
CAAACTCAAGTGTTGCCTGGTCCGTTACCTCCAGAGGCTCTGTGTCCCTCTGTTAGGTAGGGCTGACCCAGTGTC
TGGGATCCAGGTGAGACATTACCAGACTCCCCTGGCCTGTCTGTATGAATGTATGGTGACCACCCCTGCTTGTCT
TTACGTGTGCCTTTCTATATGATTCTGTCAGTTCTATAGACTGGAGAAACATCGAAAGTAGAAAGGGAACAGTC
                                                   rCyp2d-down_Large_R
```

Supplementary Figure 22. gRNAs designed to target upstream of the rat *Cyp2d2* locus and downstream of the rat *Cyp2d4* locus. The gRNA-binding sequences (blue) and the PAM sequences (green) are shown on the rat *Cyp2d* cluster sequences (yellow and green boxes). The ssODN sequences are underlined (navy). The primer sets (small and large) used for the PCR analysis are shown in boxes (see **Supplementary Table 6**).

CRISPR-mediated indel mutations at rat *Cyp2d-up and Cyp2d-down* loci

			rCyp2d-up			***rCyp2d-down***
Wistar	WT		ACCTACCGTCTCTTCAGGGTAACTGTGGTTTTA	WT		AGACCTAACCCATCAAATTCTATCCTGGGAAGG
#1	KI/KO	-9	ACCTACCGTCTCTTCAG---------GGTTTTA	KI/KO	+1	AGACCTAACCCATCAAATTCTATTCCTGGGAAGG
#2	KO	-2	ACCTACCGTCTCTTCAGGGTAA--GTGGTTTTA	KO	+1	AGACCTAACCCATCAAATTCTACTCCTGGGAAGG
#3	KO/WT	+1	ACCTACCGTCTCTTCAGGGTAAACTGTGGTTTTA	KO/WT	-6	AGACCTAACCCATCAA------TCCTGGGAAGG
#4	KO	-6	ACCTACCGTCTCTTCAGG------GTGGTTTTA	KO/WT	+4	AGACCTAACCCATCAAATTCTATTTTTCCTGGGAA
		-8	ACCTACCGTCTCTTCA--------GTGGTTTTA			
#5	KO	-6	ACCTACCGTCTCTTCAGG------GTGGTTTTA	KO/WT*	+1	AGACCTAACCCATCAAATTCTATTCCTGGGAAGG
					-5	AGACCTAACCCATCAAATT-----CTGGGAAGG
#6	KO/WT	-75	ACCTACCGTCTCTTCAGGGT--(-75bp)--AAGAA	KO/WT	+1	AGACCTAACCCATCAAATTCTAATCCTGGGAAGG
#7	KO	-6	ACCTACCGTCTCTTCAGG------GTGGTTTTA	KO	+1	AGACCTAACCCATCAAATTCTACTCCTGGGAAGG
		-8	ACCTACCGTCTCTTC--------TGTGGTTTTA		-4	AGACCTAACCCATCAAAT----TCCTGGGAAGG
#8	KO	-8	ACCTACCGTCTCTTC--------TGTGGTTTTA	KO	+1	AGACCTAACCCATCAAATTCTACTCCTGGGAAGG
#9	KO	+1	ACCTACCGTCTCTTCAGGGTAAACTGTGGTTTTA	KO/WT	+1	AGACCTAACCCATCAAATTCTACTCCTGGGAAGG
		+3	ACCTACCGTCTCTTCAGGGTAGTTTCTGTGGTTTTA			
#10	KO/WT	-9	ACCTACCGTCTCTTCAG---------GGTTTTA	KO	-8	AGACCTAACCCATCAAATTT--------GAAGG
#11	KO/WT	-6	ACCTACCGTCTCTTCAGG------GTGGTTTTA	KO	-3	AGACCTAACCCATCAAATTC---CCTGGGAAGG
#12	KO	-9	ACCTACCGTCTCTTCAG---------GGTTTTA	KO/WT	+1	AGACCTAACCCATCAAATTCTACCCCTGGGAAGG
		+2	ACCTACCGTCTCTTCAGGGTAAAACTGTGGTTTTA			
#13	KO	-8	ACCTACCGTCTCTTC--------TGTGGTTTTA	KO	+1	AGACCTAACCCATCAAATTCTACTCCTGGGAAGG
		+14	ACCTACCGTCTCTTCAGGGTAAGA(+14bp)TCCTGT		+2	AGACCTAACCCATCAAATTCTATTTCCTGGGAAGG
#14	KO/WT	-8	ACCTACCGTCTCTTC--------TGTGGTTTTA	KO/WT*	+1	AGACCTAACCCATCAAATTCTACTCCTGGGAAGG
					-14	AGACCTAA--------------TCCTGGGAAGG
#15	KO/WT*	-2	ACCTACCGTCTCTTCAGGGT--CTGTGGTTTTA	KO/WT*	-1	AGACCTAACCCATCAAATTCT-TCCTGGGAAGG
		-9	ACCTACCGTCTCTTCAG---------GGTTTTA		+2	AGACCTAACCCATCAAATTCTATTTCCTGGGAAGG
#16	KO	-8	ACCTACCGTCTCTTCA--------GTGGTTTTA	WT	w	
#17	KO/WT	-6	ACCTACCGTCTCTTCAGG------GTGGTTTTA	KO/WT*	-1	AGACCTAACCCATCAAATTCTAT-CTGGGAAGG
					-4	AGACCTAACCCATCAAATTCTAT----GGAAGG
#18	KO	-1	ACCTACCGTCTCTTCAGGGTT--CTGTGGTTTTA	KO	+1	AGACCTAACCCATCAAATTCTATTCCTGGGAAGG
#20	KO/WT	-9	ACCTACCGTCTCTTCAG---------GGTTTTA	KO/WT	-7	AGACCTAACCCATCAAATTCTAG-------AGGTC
#21	KO	-9	ACCTACCGTCTCTTCAG---------GGTTTTA	KO/WT*	+1	AGACCTAACCCATCAAATTCTACTCCTGGGAAGG
					+4	AGACCTAACCCATCAAATTCTATTTTTCCTGGGAA
#22	KO	-10	ACCTACCGTCTCTTCA----------GGTTTTA	KO	+1	AGACCTAACCCATCAAATTCTATTCCTGGGAAGG
					+19	AGACCTAACCCATCAAATTCTAAT(+19bp)ATTCCT
#23	KO	-6	ACCTACCGTCTCTTCAGG------GTGGTTTTA	KO	-1	AGACCTAACCCATCAAATTCT-TCCTGGGAAGG

Supplementary Figure 23. NHEJ-mediated KO mutations at rat *Cyp2d* cluster sites. Cas9 mRNA and three gRNAs; one targeting the upstream of rat *Cyp2d2*, the second targeting downstream of rat *Cyp2d4*, and the third on the plasmid DNA, together with two ssODNs and the human *CYP2D6* plasmid were microinjected into the male pronucleus of fertilized Wistar rat embryos. Sequence analysis of the 23 pups delivered showed a variety of indel mutations at the targeted *Cyp2d2* and *Cyp2d4* loci, as shown by red letters (KO). Asterisks indicate pups carrying mosaic mutations.

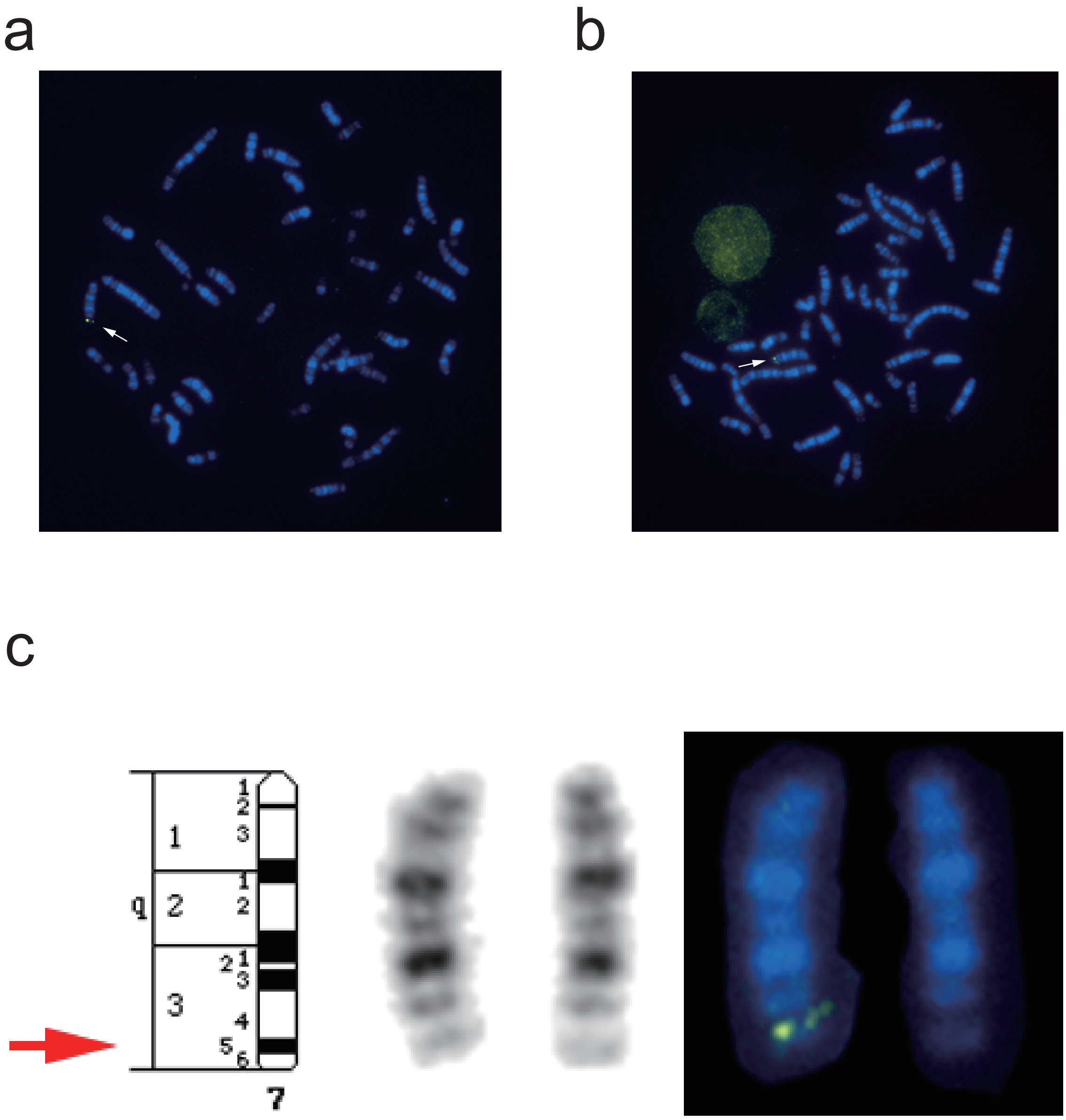

Supplementary Figure 24. Fluorescent in situ hybridization (FISH) analysis of the homozygous CYP2D6-KI rats. CYP2D6-KI allele hybridized with the fluorescent labeled probes (arrows) (a, b). The presence of the heterozygous KI allele was confirmed on rat chromosome 7q34 (c).

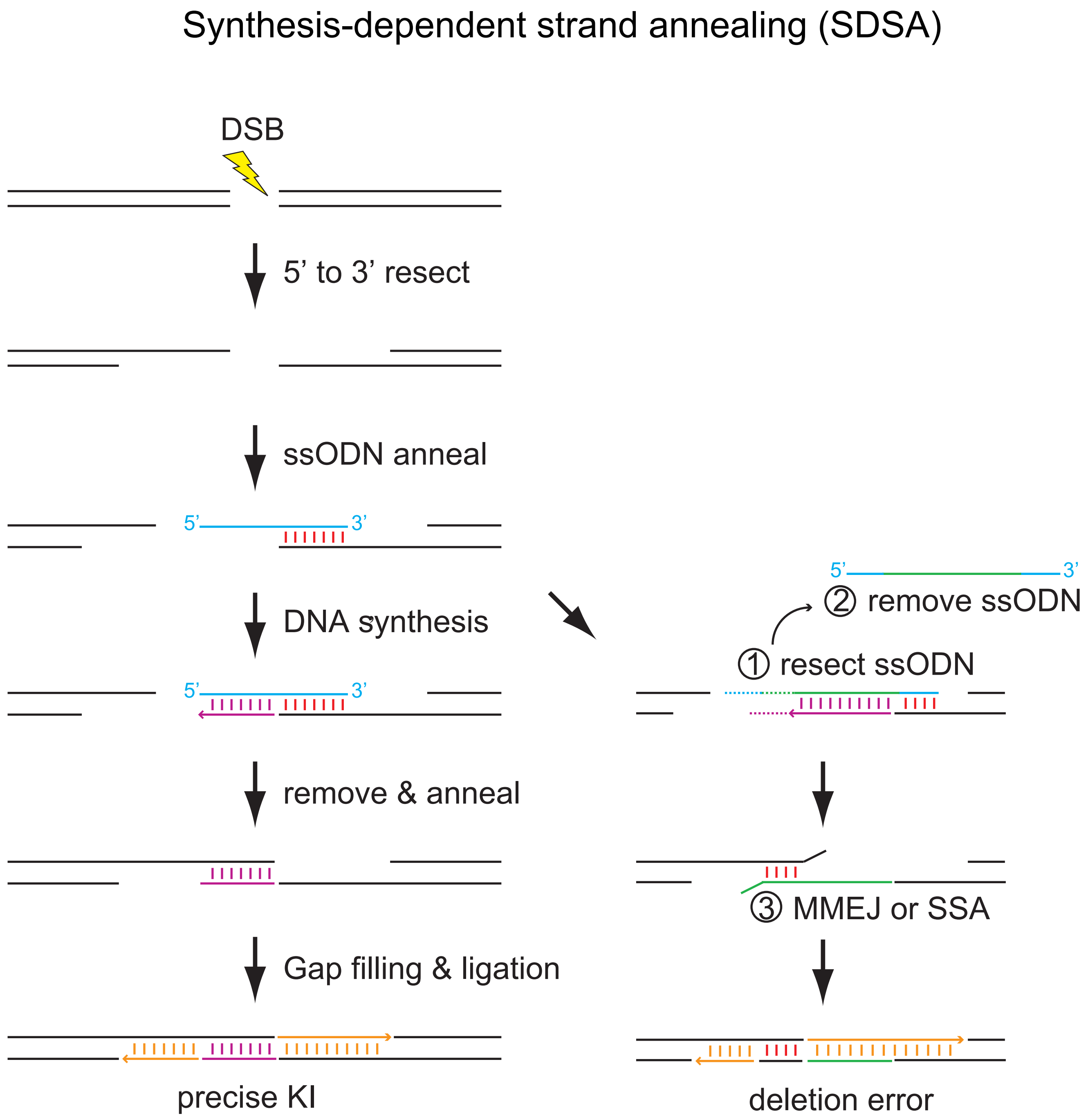

Supplementary Figure 25. Hypothesis of the HDR mechanism. In the synthesis-dependent strand annealing (SDSA) pathway, the 3' end of an ssODN anneals to the homologous sequences on the 3' strand of the DSB after resection of the 5' strand of the DSB. DNA synthesis occurs from the 3' end of the DSB to copy the 5' remaining strand of the ssODN. A second annealing occurs with the other 3' end of the DSB after removal of the ssODN. Finally, gap filling and ligation complete the repair event. In the pathway, (1) resection of 5' ssODN by exonuclease activity, or (2) removal of the ssODN by helicase activity concurrently occurs with DNA synthesis, which causes incomplete repair, such as indel mutations. (3) Microhomology sequences between the 3' strand of the DSB and the 3' of the newly synthesized strand from the ssODN may also cause indel mutations via microhomology-mediated end joining (MMEJ) or single-strand annealing (SSA).

Supplementary Table 1. Comparison of genome editing efficiency between Cas9 and Cas9-polyA in rats using the 2-cell embryo assay.

Targeted locus	Injected RNA	Embryos injected	2-cell embryos (%)	PCR-amplified (%)	Knockout (%)
Tyr	Cas9-GFP	29	24 (82.8)	20 (83.3)	2 (10.0)
	Cas9-GFP-polyA	35	24 (68.6)	19 (79.2)	7 (36.8)*

Two-cell embryos were collected 24 h after injection of Cas9 mRNA/gRNA, and genomic DNA was amplified by PCR to detect NHEJ-mediated knockout mutations at the targeted locus. *Tyr*: tyrosinase, *$P<0.05$, Cas9 vs. Cas9-polyA by Fisher's exact test.

Supplementary Table 2. Potential off-target sites for CRISPR/Cas targeting in the rat genome.

Name	Sequence	Hit Score*	No. of Mismatches	Coordinate (rn5)	Mutation Frequency (Mutant/Total)	Primer Forward	Primer Reverse
**gRNA:*Tyr*^{c}**	**TTTCCAGGATTATGTAATA GTGG**						
Tyr^{c}-OT1	CTCCCAGGGTTATGTAATAG AAG	1.6	3	chr19:+32965297	0/13	TTGAATCCTGTGATACAGTTCAG AC	CATATGCCGAGGTCACAACA
Tyr^{c}-OT2	TATCCAGGCATATGTAATAG CAG	1.5	3	chr10:-32914681	0/13	TGGCCATACTCCAAATCTTCTT	CCCTGATCCTGTAGTTTTGT GA
Tyr^{c}-OT3	ATTCCAGGAGTATGTAATAC TAG	1.4	4	chr19:+24325208	0/13	GGACACCTGCAGTAAGGACA	TGAGATTGAGATCATTTTGG TCA
Tyr^{c}-OT4	TTTCCAGAACTATGTAATAC TGG	1.1	3	chr11:+34484150	0/13	GAGTGAGCAATGGCGGATAC	CACAGTTGCTCACACGCTTT
Tyr^{c}-OT5	TTTCCATGTGTATGTAATAG AAG	0.9	3	chr4:+22739080	0/13	TGTGGTATCAGCTGGAGCAT	CAGAGCAGATGGCACTCCTA
Tyr^{c}-OT6	TTACTATTATTATGTAATAG TAG	0.9	4	chr10:-78685738	0/13	AGGCGAGGATGTATTGGTTG	CATGCATGTGAAGCAGGACT
Tyr^{c}-OT7	TTGCTATGACTATGTAATAG GAG	0.9	4	chr13:-118286168	0/13	TCAACCACAGGTTTGGGAAT	CTCTTGTTCATGGCCTCTCC
**gRNA:Thy1-ATG**	**GATGCTGATGACTGGGTTC ATGG**						
Thy1-ATG-OT1	GAAGCTGATGGCTGGGTTCA TGG	4.1	2	chr1:+95817250	0/8	ACCAGGTCACAGGGCTACTG	CAAGCTGGGCGAACTCTCTA
Thy1-ATG-OT2	GACTATGTTGACTGGGTTCA AAG	1.3	4	chr1:-203590002	0/8	GCCTAGTGAGCCCAGACAGT	AGCGACATGTACAACCACCA
Thy1-ATG-OT3	GATGCTAATGACTGGGTGCA CAG	1.2	2	chr1:+104389339	0/8	CTGACTGGGCATCACCATTT	GACCAGTTTGGCCTTTAGGA
Thy1-ATG-OT4	TATGCTAATGTCTGGGTTCA AAG	1.1	3	chr5:-155608310	0/8	CAGAGTGGGGAAGCCATTTA	ACATTACTGGGCCACACTCC
Thy1-ATG-OT5	GAATATTATGACTGGGTTCA TAG	0.9	4	chr2:-99063815	0/8	CCCAGCCCCACGTATATTTT	AAGAGCTCTGGGTCCCATTT
Thy1-ATG-OT6	TAAGCTTATCACTGGGTTCA CAG	0.9	4	chr19:-54038098	0/8	TGTCAATCTCCCTCGCTTCT	TTGCAATGTCCCAGGTTACA
Thy1-ATG-OT7	GTTGCTCCTCACTGGGTTCA TAG	0.9	4	chr13:-103736046	0/8	CCGGGTTACATGAGACGAAC	CTGACAACCCACTGATCCAC T
Thy1-ATG-OT8	GCTTCTCATCACTGGGTTCA TGG	0.9	4	chr15:-64251828	0/8	CCTTGAATTCTTCCAAAGCAG	CACAACCATGCACACATGAA
**gRNA:Thy1-TGA**	**GGACTTCATTTCTCTGTGA CTGG**						
Thy1-TGA-OT1	GAATTTCTTTTCTCTGTGAC AGG	2.5	3	chr3:-94266024	0/8	CACACACACACACGTGAACAA	TTGATGAGAGGTTGGTCTTT G
Thy1-ATG-OT2	GAAATTCATATCTCTGTGAC TGG	2.5	3	chr9:+72220986	0/8	GGTTTGAACTTTGGGCTTTG	TGTAGGGGGTGCTAGAGTGG
Thy1-ATG-OT3	GGACATTCTTTCTCTGTGAC TGG	1.6	3	chr2:-223297766	0/8	CCCACAGAAAATACACGCAAA	ACACAGAGGCTGCAGGACTT
Thy1-ATG-OT4	GCTCTTCAGTTCTCTGTGAC TGG	1.6	3	chr8:-75966086	0/8	CGGGAAAAAGCACTGGTAAA	TACCAAGTGGCTCACAACCA
Thy1-ATG-OT5	TATCATCATTTCTCTGTGACT GG	1.3	4	chr1:+159654698	0/8	TCCATTTGCATGTGTTTGGT	TGTGAATGGAGCTGGCTTAG
Thy1-ATG-OT6	TGACTTCCTTTCTCTGGGAC CAG	1.3	3	chr14:-82502071	0/8	GTAGATGGCCAGGGATAGCA	TGCAGGATTCTGACAAGTGG
Thy1-ATG-OT7	GGATTTCATTTCTTTGTGAC CAG	1.3	2	chrX:+54020076	0/8	CGTTCTTGTTCCCCTTGTGT	TTTGTTTGCCTGTATGTTGGA
Thy1-ATG-OT8	TTACTTCATTTCTCTGTGGCA	1.1	3	chr1:-36741643	0/8	TCAACAGTCTCTGCCTCTGC	CCTTGGCCTGCATAAATGTT

	GG						
Thy1-ATG-OT9	GAACTGTATTTCTCTGTGACCAG	1	3	chr1:-275694272	0/8	GAGGACCTTGAGGCAGAGTG	CAGCTGGGGAGACAAAAGAG
Thy1-ATG-OT10	AGACTGCAGTTCTCTGTGACCAG	1	3	chr16:+63188060	0/8	AGAACTCCATGGCAGATGGT	TGCTGTAAGGGGTGTGTGTG
Thy1-ATG-OT11	GAACTACACTTCTCTGTGACCAG	1	3	chr2:+143964172	0/8	AATGGTCAAAATGCCTGAGC	TCAACTGTTGCAAAAGGGAGT
**gRNA:rRosa26**	**GACTCCAGTTGCAGATCACGAGG**						
rRosa26-OT1	GACCCCAGTTGCAGATCACAGGG	6.4	2	chr6:-146497662	0/4	CACGACCCCTACCATTCATT	ATTGTGGCCTTACCTGGTTG
rRosa26-OT2	GACCCCAGTTGCAGAGCACGGAG	1.7	2	chr18:+55462095	0/4	CTGAAGGTAACCCGTCCTCA	CTGTGCCCTCATCGTCCTAT
rRosa26-OT3	GCCTGCAGTTGCAGATCACAAGG	1.5	3	chr5:+172749987	0/4	GCTGCCACTACTGTGTCCAG	TCATTGTGGAGTCCCTGACC
rRosa26-OT4	GATTCCAGCAGCAGATCACGTGG	1.4	3	chr14:-78446863	0/4	GCAAGAGCCTGAAATCCTCA	TCTTCCTCGGTGACTGTCCT
rRosa26-OT5	ATCATCAGTTGCAGATCACGTGG	1.3	4	chr5:-30258553	0/4	TAAGGCCACTGGAAGACTGG	AGACCAACTCGGTTTGCATC
rRosa26-OT6	GGCTCCTGTTGAAGATCACGAAG	0.9	3	chr3:+110807013	0/4	TCACATGGGAAAGTCCATGA	CCCTCTCCCTTCTTCCTTTC
rRosa26-OT7	GATTGCTGTAGCAGATCACGGGG	0.9	4	chr19:-71341543	0/4	TTGCTAAACTGGGCTTCAGG	CTCACTTGAGGGGCTTTGAG
rRosa26-OT8	ACCTCCATTTCCAGATCACGGGG	0.8	4	chr3:+141310868	0/4	CGTCACGTCAGCAGTCAGTAA	ACAAGCACCCTTACCTGTCG
**gRNA:CAGGS**	**CAGGGTTATTGTCTCATGAGCGG**						
pCAG-OT1	CCTGATTATTGTCTCATGAGAAG	2.3	3	chr13:-43521965	0/4	TCTTAACATGCTGCCCCTTC	TGAAAAAGCCATCAGTTGGTC
pCAG-OT2	TAGTGTAATTGTCTCATGAGAAG	1.7	3	chr6:+3958889	0/4	TGCACTCTTGGCATAAGACG	CCAGGCTTCCATTGCTACTC
pCAG-OT3	GAGGATTATTATCTCATGAGGAG	1.6	3	chr4:+147313287	0/4	GGATTCGATCCTTAGCACCA	TGACAAAGGGTTGTGTGCAT
pCAG-OT4	CATTGTTATTTTCTCATGAGAAG	1.5	3	chr5:-77761908	0/4	GGGACAGACAGAAAGAGATACCA	GGGAAGCAGAGCAAAGAATG
pCAG-OT5	TTGCGTTGTTGTCTCATGAGTAG	1.4	4	chr13:+81796711	0/4	CATACCGTGGCACATGAACT	AAAACCCCCAATGCTTTTTC
pCAG-OT6	TCTGATTATTGTCTCATGAGAAG	1.3	4	chr2:-142513379	0/4	TCGGACCTAGACACCTTTCC	TCCCTGCAGAGAAGAAGCAT
pCAG-OT7	GAAGGTTTTCGTCTCATGAGTAG	1.3	4	chr7:+142228609	0/4	TGGCACCCATTCTTGAACAT	AGGGGACACCAGTGACTTTG
pCAG-OT8	TGCTGTTATTGTCTCATGAGTAG	1.3	4	chr1:-224044123	0/4	CTCCCTTCTCCCTCCAAACT	ACTCTTGCCACTGTGCCTTT
pCAG-OT9	GAGAGTATTTGTCTCATGAGAAG	0.9	4	chr11:-60248423	0/4	TTCAAGACCATGCACAGGAG	ATGCAATGTCCAAGCAATGA

*Off-target hit scores are calculated by CRISPR Design Tool (http://crispr.mit.edu/)

Supplementary Table 3. CRISPR/Cas-mediated knock-ins in rats.

Embryos injected	Two-cell embryos (%)	Pups delivered (%)	Knockouts /offspring (%)	Knock-ins /offspring (%)
145	88 (60.7)	48 (54.5)	37/48 (77.1)	5/48 (13.5)

Cas9-poly(A) mRNA, gRNA:Thy1-ATG, and lsODN-ATG(+) were injected into fertilized rat eggs. Two-cell embryos were transferred into pseudopregnant female rats and delivered offspring were genotyped by PCR and sequencing to detect knockout (KO) or knock-in (KI) alleles (See **Fig. 2c, d**).

Supplementary Table 4. Germline transmission from offspring generated by CRISPR/Cas.

KI rat	Founder No.	CRISPR-mediated mutation	G1 offspring	G1 mutants
CAG-GFP	8	Tg	16	8
CAG-GFP	11	KI	16	8
hCYP2D6	8	Tg	3	2
hCYP2D6	2	Large Del	9	3
hCYP2D6	18	KI	17	8

Supplementary Table 5. CRISPR/Cas-mediated knock-ins in rats.

Embryos injected	Two-cell embryos (%)	Pups delivered (%)	Knockouts /offspring (%)	Knock-ins /offspring (%)
100	56 (56.0)	21 (37.5)	20/21 (95.2)	6/21 (28.6)

Cas9-poly(A) mRNA, gRNA(s), ssODN-1(-) upstream and ssODN-2 (+) downstream of the rat *Rosa26* cut site, and a donor plasmid were injected into fertilized rat eggs. Two-cell embryos were transferred into pseudopregnant female rats and delivered offspring were genotyped by PCR and sequencing to detect knockout (KO) or knock-in (KI) alleles (See **Supplementary Fig. 14**).

Supplementary Table 6. Primers used for mutation screening.

Primer name	Forward	Reverse	PCR size (bp)
rTyr Small	TTGCATAAATTGGTTTTCACAGA	ATTTAAACATGAAAATATTACCTTCCA	260
rThy1-ATG Small	GAGGTGTCCATTGTGTGATCC	CCCGAGTGTCTGAGACTATTGC	253
rThy1-TGA Small	GGTTGGTACCCCTTCTCTCC	GACAGGGTCCCCATTTCTC	266
EGFP Small	CTACCCCGACCACATGAAG	CTTGTGCCCCAGGATGTT	202
rRosa26 Small	AAGGGAGCTGCAGTGGAGTA	CCCAGGTGAGTGCCTAGTCT	360
rRosa26 Large	ATTGGCTCTCGGGGCTCAGAAAACT	AGGAGAGAGGGAATGCCAGTGC	1061
mRosa26 Small	AAGGGAGCTGCAGTGGAGTA	CCGAAAATCTGTGGGAAGTC	297
mRosa26 Large	ATTGGCTCTCGGGGCCCAGAAAACT	AGGAAAGGGAAAATGCCAATGC	1072
CAGGS Small	ACTTTCACCAGCGTTTCTGG	AATCAATGTCGACCCAGGTG	237
CAGGS Large	TCAAGGCGAGTTACATGATCC	GCCAAGTAGGAAAGTCCCATAA	962
rSirpa Exon2 Small	GCTGCCATCTTTCTCTCCAG	CAGATGCTAAAGTCCATATTGTTTC	261
rSirpa Exon2 Large	TTCAGGCTCCCATTTTTGTC	GGCATTCGATGCAGCATTAT	1613
PISceI Small	GGCAGTTATTGGTGCCCTTA	TTCAACCCAGTCAGCTCCTT	353
PISceI Large	TCCGGCCTTTATTCACATTC	GGGAAACGCCTGGTATCTTT	1557
hSIRPA up Small	AGGTCTTACTGGCCCCCTTA	TCTGTGCCTCAGCTTCTTCA	374
pBACe3.6 Small	CTTGTTGGGCGATAATCGTT	TACTGAGGACGCACTGGATG	165
hSIRPA Exon1 Small	GGGGGAGCCTTAGTCATTTC	AGGCGTCTCTCCCCCTATTA	287
hSIRPA Exon8 Small	CAGATGTCGCAAGCCCTATT	CTGTCCACATGGTCTGTTGG	263
rSirpa cDNA	AATGTCACCCCAGAAGATGC	TCCGTCCGTGATAAGTTTCC	563
hSIRPA cDNA	GCCGGCACCTACTACTGTGT	GGGCTGTTGAGTAACCTCCA	441
rCyp2d up Small	GGTCACCTCCTCTCCATGTG	GCTAGGAGTCCAGGTGCTTG	274
rCyp2d up Large	GACCTCCCACACCTGTCTGT	CTTTCTTCTCTCCGGTGGTG	1288
rCyp2d down Small	CCATTTGGGCCATAAAACTT	GCTGGCTGGTGACTACACTG	253
rCyp2d down Large	ATGCCAACATCTGGAAGGAG	GACTGTTCCCTTTCTACTTTCG	1244
hCYP2D6 Exon1	TGGCATGAAGGACTGGATTT	AAGGCCTTTCCTTCTGGTGT	153

gRNA

Tyr	TAGGTTTCCAGGATTATGTAATAG	AAACCTATTACATAATCCTGGAAA
rThy1-ATG	TAGGGATGCTGATGACTGGGTTCA	AAACTGAACCCAGTCATCAGCATC
rThy1-TGA	TAGGGGACTTCATTTCTCTGTGAC	AAACGTCACAGAGAAATGAAGTCC
rRosa26	TAGGGACTCCAGTTGCAGATCACG	AAACCGTGATCTGCAACTGGAGTC
mRosa26	TAGGGACTGGAGTTGCAGATCACG	AAACCGTGATCTGCAACTCCAGTC
pCAGGS	TAGGCAGGGTTATTGTCTCATGAG	AAACCTCATGAGACAATAACCCTG
rSirpa	TAGGGTGTCCTCCCTGACGCCTGT	AAACACAGGCGTCAGGGAGGACAC
PISceI	TAGGTATGTCGGGTGCGGAGAAAG	AAACCTTTCTCCGCACCCGACATA
rCyp2d up	TAGGCCGTCTCTTCAGGGTAACTG	AAACCAGTTACCCTGAAGAGACGG
rCyp2d down	TAGGTAACCCATCAAATTCTATCC	AAACGGATAGAATTTGATGGGTTA
**ssODN**		
Tyr[C]	AGGTTTTATGTGATGGAACACCTGAGGGACCACTATTACGTAATCCTGGAAACCATGACAAAGCCAAAACCCCCAGGCTC	
rRosa26-CAGGS	CCCTGGGCCTGGAAGATTCCCTTCCCCCTTCTTCCCTCGTGAGCGGATACATATTTGAATGTATTTAGAAAAATAAACAA	
rRosa26-CAGGS (-)	TTGTTTATTTTTCTAAATACATTCAAATATGTATCCGCTCACGAGGGAAGAAGGGGGAAGGGAATCTTCCAGGCCCAGGG	
CAGGS-rRosa26	TTCAATATTATTGAAGCATTTATCAGGGTTATTGTCTCATGATCTGCAACTGGAGTCTTTCTGGAAGATAGGCGGGAGTC	
mRosa26-CAGGS	GCCCTGGGCCTGGGAGAATCCCTTCCCCCTCTTCCCTCGTGAGCGGATACATATTTGAATGTATTTAGAAAAATAAACAA	
CAGGS-mRosa26	TTCAATATTATTGAAGCATTTATCAGGGTTATTGTCTCATGATCTGCAACTCCAGTCTTTCTAGAAGATGGGCGGGAGTC	
rSirpa-PISceI	AGTGTCTGTTGCTGCTGGAGATTCGGCCACTCTGAACTGCACTGTGTCCTCCCTGACGCCAAGAGGTAATGAAATGTGCCCCGCTTACGCAGGGCATCCATTTATTAC TCAACCGTAACC	
PISceI-rSirpa	ACTAGATAACTTCGTATAATGTATACTATACGAAGTTATTATCTATGTCGGGTGCGGAGATGTGGGACCCATTAAGTGGTTCAAAGGAGAAGGGCAAAATCGGAGCC CGATCTACAGTTT	
rCyp2dUp-CAGGS	CTAGTGACAGGGCCTGGTGCCCAGGAGTCAGGCAAAACACCTACCGTCTCTTCAGGGTAAGAGCGGATACATATTTGAATGTATTTAGAAAAATAAACAAATAGGGG TTCCGCGCACATT	
CAGGS-rCyp2dDown	AATACTCATACTCTTCCTTTTTCAATATTATTGAAGCATTTATCAGGGTTATTGTCTCATTCCTGGGAAGGTCTCTTGAAGCACTCATCTTGGCTTCTTGGCTTCTGTAG TTCTCCTAGC	
